# Metabolic transcription analysis of engineered *Escherichia coli *strains that overproduce L-phenylalanine

**DOI:** 10.1186/1475-2859-6-30

**Published:** 2007-09-19

**Authors:** José Luis Báez-Viveros, Noemí Flores, Katy Juárez, Patricia Castillo-España, Francisco Bolivar, Guillermo Gosset

**Affiliations:** 1 Centro de Investigación en Biotecnología, Universidad Autónoma del Estado de Morelos, Av. Universidad 2000, Cuernavaca, Morelos, México; 2 Departamento de Ingeniería Celular y Biocatálisis, Instituto de Biotecnología, Universidad Nacional Autónoma de México, Av. Universidad 2001, Cuernavaca, Morelos, México

## Abstract

**Background:**

The rational design of L-phenylalanine (L-Phe) overproducing microorganisms has been successfully achieved by combining different genetic strategies such as inactivation of the phosphoenolpyruvate: phosphotransferase transport system (PTS) and overexpression of key genes (DAHP synthase, transketolase and chorismate mutase-prephenate dehydratase), reaching yields of 0.33 (g-Phe/g-Glc), which correspond to 60% of theoretical maximum. Although genetic modifications introduced into the cell for the generation of overproducing organisms are specifically targeted to a particular pathway, these can trigger unexpected transcriptional responses of several genes. In the current work, metabolic transcription analysis (MTA) of both L-Phe overproducing and non-engineered strains using Real-Time PCR was performed, allowing the detection of transcriptional responses to PTS deletion and plasmid presence of genes related to central carbon metabolism. This MTA included 86 genes encoding enzymes of glycolysis, gluconeogenesis, pentoses phosphate, tricarboxylic acid cycle, fermentative and aromatic amino acid pathways. In addition, 30 genes encoding regulatory proteins and transporters for aromatic compounds and carbohydrates were also analyzed.

**Results:**

MTA revealed that a set of genes encoding carbohydrate transporters (*galP*, *mglB*), gluconeogenic (*ppsA*, *pckA*) and fermentative enzymes (*ldhA*) were significantly induced, while some others were down-regulated such as *ppc*, *pflB*, *pta *and *ackA*, as a consequence of PTS inactivation. One of the most relevant findings was the coordinated up-regulation of several genes that are exclusively gluconeogenic (*fbp*, *ppsA*, *pckA*, *maeB*, *sfcA*, and glyoxylate shunt) in the best PTS^- ^L-Phe overproducing strain (PB12-ev2). Furthermore, it was noticeable that most of the TCA genes showed a strong up-regulation in the presence of multicopy plasmids by an unknown mechanism. A group of genes exhibited transcriptional responses to both PTS inactivation and the presence of plasmids. For instance, *acs-ackA*, *sucABCD*, and *sdhABCD *operons were up-regulated in PB12 (PTS mutant that carries an *arcB*^- ^mutation). The induction of these operons was further increased by the presence of plasmids in PB12-ev2. Some genes involved in the shikimate and specific aromatic amino acid pathways showed down-regulation in the L-Phe overproducing strains, might cause possible metabolic limitations in the shikimate pathway.

**Conclusion:**

The identification of potential rate-limiting steps and the detection of transcriptional responses in overproducing microorganisms may suggest "reverse engineering" strategies for the further improvement of L-Phe production strains.

## Background

**Figure 1:**
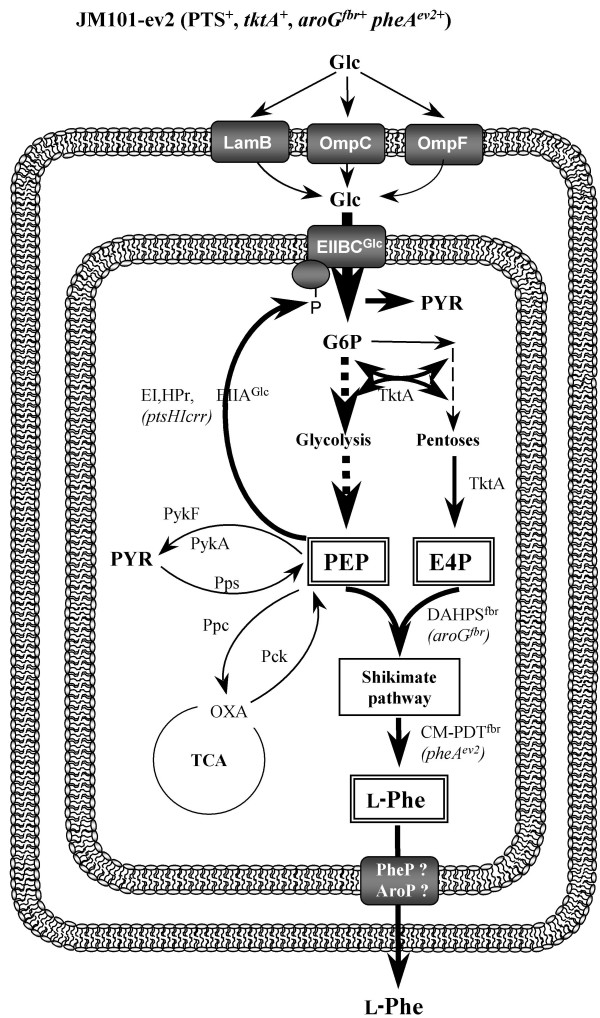
**Glucose transport and central metabolism reactions in *E. coli *strains with an active PTS**. Glucose transport through outer and inner membranes and main pathways involved in the biosynthesis of L-Phe in strains with an active PTS (strain JM101).

**Figure 2:**
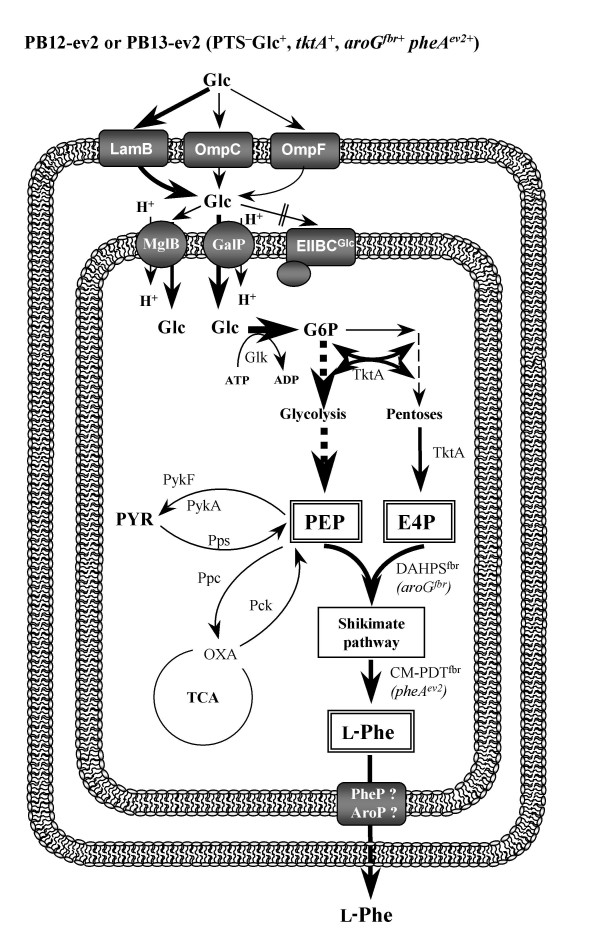
**Glucose transport and central metabolism reactions in *E. coli *strains with an inactive PTS**. Glucose transport through outer and inner membranes in derivative *E. coli *strains with an inactive PTS. These strains such as PB12-ev2 and PB13-ev2 use GalP and Glk for glucose transport and phosphorylation.

Metabolic engineering is the specific modification of the metabolic pathways or the introduction of new ones within the host organism by means of genetic engineering techniques [1]. In the context of L-phenylalanine (L-Phe) production, the challenge to design and construct L-Phe overproducing strains has been approached by using several genetic strategies: 1) the deregulation and overexpression of key enzymes. For example 3-deoxy-D-*arabino*-heptulosonate 7-phosphate (DAHP) synthase and chorismate-mutase prefenate-dehydratase (CM-PDT), are two essential steps to overcome these metabolic bottlenecks that strongly control the carbon flux directed into the biosynthesis of L-Phe. 2) When these rate-limiting steps have been overcome, additional strategies are necessary to increase the availability of precursors for aromatic biosynthesis: phosphoenolpyruvate (PEP) and erythrose 4-phosphate (E4P). Some of these genetic strategies have been successfully applied, achieving the purpose of increasing PEP and E4P availability [2-5]. In general, these consist of inactivating enzymes that consume PEP and/or overexpressing enzymes that produce E4P and/or PEP. For instance, the overexpression of either transketolase (*tktA*) or transaldolase (*talA*) combined with the overexpression of feedback insensitive DAHP synthase increased the synthesis of aromatic compounds in *E. coli *strains, presumably by increasing E4P availability [2,6,7]. Alternatively, the overexpression of PEP synthase (*ppsA*) in *E. coli *augmented PEP availability, and thereby, the yield in the synthesis of aromatic compounds from glucose [8]. Likewise, the inactivation of PEP carboxylase (*ppc*) or pyruvate kinases (*pykA*, *pykF*) also led to an increase in PEP availability [9-11]. The inactivation of the main glucose transport system, known as phosphoenolpyruvate: carbohydrate phosphotransferase system (PTS) has shown a great impact on PEP availability, increasing substantially the biosynthetic capacity of aromatic compounds [2,11-16]. The construction of PTS mutants (PTS^-^Glc^-^) has been reported and from these strains spontaneous PTS^-^Glc^+ ^mutants were selected, which have an enhanced capacity to transport glucose [2]. Briefly, deletion of the PTS operon (*ptsHIcrr*) in strain JM101 (μ = 0.71 h^-1^) generated strain PB11 (PTS^-^), which grows slowly in minimal media supplemented with glucose (μ = 0.1 h^-1^). The PB11 mutant was subjected to an adaptive evolution process in which spontaneous PB12 (μ = 0.42 h^-1^) and PB13 (μ = 0.49 h^-1^) mutants were isolated, showing a significantly higher specific growth rate on glucose (PTS^-^Glc^+ ^phenotype) than in PB11 [2]. Further characterization of PB12 and PB13 mutants showed that glucose is mainly internalized into the cell by the galactose permease (GalP) and phosphorylated by glucokinase (Glk) [17,18] (Figures 1, 2). At least two spontaneous mutations occurred when PB12 was selected, being one of them a mutation in *arcB *gene that is partially responsible for the up-regulation of TCA cycle genes when this strain grows on glucose as the sole carbon source [18,19]. In addition, metabolic flux analysis, using NMR, revealed that these mutants exhibit important changes in the distribution of carbon flux at the level of the central metabolism [17]. The redistribution of carbon fluxes in PTS mutants can be beneficial for the synthesis of aromatic compounds, as has been suggested by studies of the NF9 strain (PTS^-^Glc^+^) engineered to overproduce the first aromatic intermediate (DAHP) [2,12]. In the latter works, it was shown that PTS inactivation has a positive impact on the productivity and yield of DAHP from glucose. The advantage of PTS mutants for the production of aromatic compounds has been confirmed with the successful construction of L-Phe overproducing *E. coli *strains, combining the simultaneous overexpression of transketolase (*tktA*), feedback insensitive DAHP synthase (*aroG*^fbr^) and evolved feedback insensitive chorismate-mutase prefenate-dehydratase CM-PDT^ev2 ^(*pheA*^ev2^) in a PTS^-^Glc^+ ^genetic background [15]. Thus, a PTS^-^Glc^+ ^derivative carrying appropriate plasmids(PB12-ev2) showed a substantial improvement in L-Phe yield from glucose (*Y*_*Phe*/*Glc *_= 0.33 g/g) with regards to PTS^+ ^strain (JM101-ev2, *Y*_*Phe*/*Glc *_= 0.22 g/g). These yields correspond to 60 and 40% of the theoretical maximum, respectively.

Recombinant DNA technology allows the introduction of specific genetic modifications within particular metabolic pathways. However, these manipulations can trigger directly or indirectly transcriptional responses of several genes, as a consequence of the inextricable complexity and connectivity of the metabolic and regulatory networks. Accordingly, some transcriptional responses in the cell may be associated with a high degree of unpredictability, and thereby with unexpected effects sometimes opposite to those desired. For instance, the use of multicopy plasmids is a very common procedure in metabolic engineering to construct recombinant organisms. However, plasmid metabolic burden is an important factor that should be taken into account when genetically engineering a strain, because broad effects on several cellular processes have been reported. Metabolic burden imposes an extra demand on cellular processes, which in turn, generate a high demand on the syntheses of precursor molecules, causing a reduction in the specific growth rate [20]. Saturation effects could also occur on cellular machineries and/or competition effects of the overexpressed gene(s) with the host cell genes and proteins. This in turn could also decrease the transcription and translation rate of other genes and mRNAs, and accordingly, the enzyme pools (dilution effect) [21-23]. Furthermore, it has been reported that overexpression of recombinant proteins in *E. coli *triggers induction of stress responses such as heat shock and SOS/DNA damage [24]. However, despite the importance of carbon central metabolism, as far as we know, there are no reports about the effects of plasmid presence on the transcription of genes involved in this part of the cellular physiology.

Basic and applied studies of Metabolic Transcription Analysis (MTA) may be valuable, because have the potential to: 1) determine transcriptional responses to the genetic modifications introduced into the organism during the process of genetic engineering, providing a deep insight on the physiological mechanisms of the cell; 2) identify significant transcript changes of key genes related to the biosynthesis of a desired metabolite in organisms that overproduce it; 3) identify transcriptional responses to multi-copy plasmid presence in the strain engineering process; 4) associate significant transcriptional alterations with the metabolic capacity of the cell, which can be assessed in terms of yields and productivities of the desired metabolite; 5) detect potential rate limiting steps in the transcription of key genes, which may be the basis to formulate new hypothesis and genetic strategies ("reverse engineering") to further improve the microbial cell factories.

Microarray and Real Time-PCR (RT-PCR) technologies are used to generate information about transcriptional responses to different conditions. Transcriptional analysis by RT-PCR has several advantages, for example, the sensitivity and accuracy of measurements is very high because the mRNA signal is exponentially amplified. This is especially useful when detecting mRNAs that are weakly expressed. Furthermore, statistical analysis of RT-PCR data has been shown to be relatively straightforward. On the other hand, microarray technology enables genome-wide transcriptional analyses, whereas RT-PCR-based methods have a practical limit of about a couple hundred genes. Therefore, RT-PCR analyses might leave out genes responding to a specific experimental condition.

Transcriptional characterization of metabolic genes in PB11 and PB12 mutants grown on glucose as the only carbon source has been reported using RT-PCR [18,19,25]. In the current study, MTA of 116 genes involved in glycolysis (Embden-Meyerhof-Parnas), gluconeogenesis, tricarboxylic acid cycle (TCA), pentose phosphate (PP), aromatic amino acids, anaplerotic and fermentative pathways was performed. In addition, the transcription of genes encoding regulatory proteins and transporters for aromatic compounds and carbohydrates were also analyzed. These genes are involved in the metabolic and regulatory networks related to aromatics biosynthesis in *E. coli*. RT-PCR technology was chosen in this study to provide precise transcriptional data for genes from these specific segments of the cell's physiology. This work describes, for the first time, metabolic transcriptional responses to PTS inactivation and multi-copy plasmid presence in PTS^+ ^and PTS^- ^L-Phe overproducing *E. coli *strains when they are grown on glucose and yeast extract as carbon sources. These studies permitted the identification of potential rate-limiting steps in the biosynthesis of L-Phe. This knowledge may provide strong basis for the design of new hypothesis and genetic strategies, which may further improve *Y*_*Phe*/*Glc *_and productivity of L-Phe in *E. coli *strains.

## Results and Discussion

The generation of L-Phe overproducing *E. coli *strains combining genetic strategies, such as PTS inactivation and the overexpression of key genes in the L-Phe synthesis (*aroG*^fbr^, *tktA *and *pheA*^ev2^) has been reported [15]. Glucose transport through outer and inner membranes in PTS^+ ^and PTS^-^Glc^+ ^genetic backgrounds is shown in Figures 1, 2, respectively, as well as the main pathways involved in the biosynthesis of L-Phe and genetic strategies used for the construction of L-Phe overproducing strains. Even though strains PB12-ev2 and PB13-ev2 have the same PTS^-^Glc^+ ^phenotype, they accumulated different acetate and L-Phe amounts in resting cell cultures using glucose as the sole carbon source (table 1). In addition, they exhibited significant differences in terms of L-Phe yield and specific productivity. The current MTA included host strains (JM101, PB12 and PB13) and their derivatives L-Phe overproducing strains (JM101-ev2, PB12-ev2 and PB13-ev2) grown in M9 medium supplemented with glucose (10 g/L) and yeast extract (5 g/L). In additional file 1, all RT-PCR data are reported as relative gene transcription levels and have been referred to JM101 strain (reference strain). The reference to JM101 strain will be always assumed and hence omitted from now on, unless otherwise stated. A criterion was arbitrarily established in order to identify transcripts that changed significantly, as compared to the reference strain (JM101). We considered that a gene was significantly up-regulated when the transcript level measured by RT-PCR was ≥ 2 and down-regulated when it was ≤ 0.5. Genes that showed significant transcriptional changes were grouped and are described in the following sections.

**Table 1:** Accumulation of aromatic intermediates, acetate, L-Phe yields and productivities in L-Phe overproducing strains assessed in resting cell cultures containing 1 g of biomass. The results of the JM101-ev2, PB12-ev2 and PB13-ev2 strains have been previously reported [15, 69].

**Strains**	**q_Glc _**(g/gDCW·h)	**q_Acetate _**(mg/gDCW·h)	**DHS **(g/L)	**SHIK **(g/L)	**L-Phe **(g/L)	*Y*_*Phe*/*Glc*_	**q_Phe _**(mg/gDCW·h)
**JM101-ev2**	0.17 ± 0.01	70 ± 0.41	0.020 ± 0.001	0.029 ± 0.002	0.396 ± 0.025	0.22 ± 0.01	36.34 ± 2.50
**PB12-ev2**	0.12 ± 0.01	30 ± 0.22	0.054 ± 0.003	0.034 ± 0.001	0.460 ± 0.015	0.33 ± 0.01	40.60 ± 3.17
**PB13-ev2**	0.14 ± 0.01	40 ± 0.25	0.054 ± 0.004	0.034 ± 0.002	0.300 ± 0.019	0.20 ± 0.02	29.25 ± 2.50

Symbols: q_Glc_, specific consumption rate of glucose; q_Acetate_, specific consumption rate of acetate; q_Phe_, specific production rate of phenylalanine; *Y*_*Phe*/*Glc*_, yield of phenylalanine synthesized from glucose. DHS, 5-dehydroshikimate; SHIK, shikimate; gDCW, grams of dry cell weight.

### Glucose transport and phosphorylation

In wild type *E. coli *cells as JM101, glucose transport and phosphorylation is mediated by PTS (Figure 1). In contrast, PTS mutants (PB12 and PB13) utilize galactose permease (GalP) and glucokinase (Glk) to transport and phosphorylate glucose, respectively (Figure 2) [2,17]. In addition to these genes, we analyzed transcriptional responses of some genes encoding carbohydrate transporters to PTS inactivation or to plasmid presence. The *ptsG *transcript levels were 2.5- and 4-fold up-regulated in PB12 and PB12-ev2, respectively (additional file 1). The *ptsG *gene is regulated in a very complex manner by several proteins including ArcA/ArcB system, cAMP-CRP complex, Mlc, FruR and Fis [26-28]. In PB12, the up-regulation of some genes regulated by the ArcA/ArcB system may be explained by the *arcB*^- ^mutation found in this strain. PB12 exhibits the same toluidine blue sensitive growth phenotype as strains lacking ArcA or ArcB activities. Therefore, it can be assumed that the specific mutation in *arcB *selected in strain PB12 is causing ArcA to remain in a non-phosphorylated state [25]. The *galP *gene was found to be strongly up-regulated only in the PTS^-^Glc^+^mutants: PB12 (30.5-fold), PB13 (12.7-fold), PB12-ev2 (11.9-fold), PB13-ev2 (25.4-fold) (additional file 1, Figures 3, 4). This high induction of *galP *correlates well with its role in PTS^- ^strains is also supported by the direct evidence on *galP *deletion in PB12 and PB13, which impairs the cell growth on glucose [17,18]. *galP *transcription is controlled by CRP-cAMP, GalR and GalS [29]. It is assumed that *galP *transcription is induced in PTS mutants because they are capable of synthesizing galactose as autoinducer of the *gal *regulon [18]. In turn, galactose inactivates GalR and GalS repressors of the *gal *regulon [29]. In order to explore the possibility that some other genes, encoding carbohydrate transporters that are capable of internalizing glucose, could be induced in response to PTS deletion, transcripts of *mglB *and *malE *genes were measured. The *mglB *transcript level, encoding another galactose transporter, was strongly increased in PB12 (58.9-fold), PB13 (6.7-fold), JM101-ev2 (2.5-fold), PB12-ev2 (119.9-fold) and PB13-ev2 (68.8-fold) (additional file 1). These results suggest that the product of this gene could have a role in glucose transport in the PTS^- ^strains. The *malKFGE *operon encodes a maltose transporter system, where the maltose binding-protein is the product of the *malE *gene. The transcript levels of *malE *were up-regulated in PB12-ev2 (25.2-fold) and PB13-ev2 (14.4-fold). All these genes were also up-regulated in PB12 when growing on glucose as the only carbon source [18].

**Figure 3:**
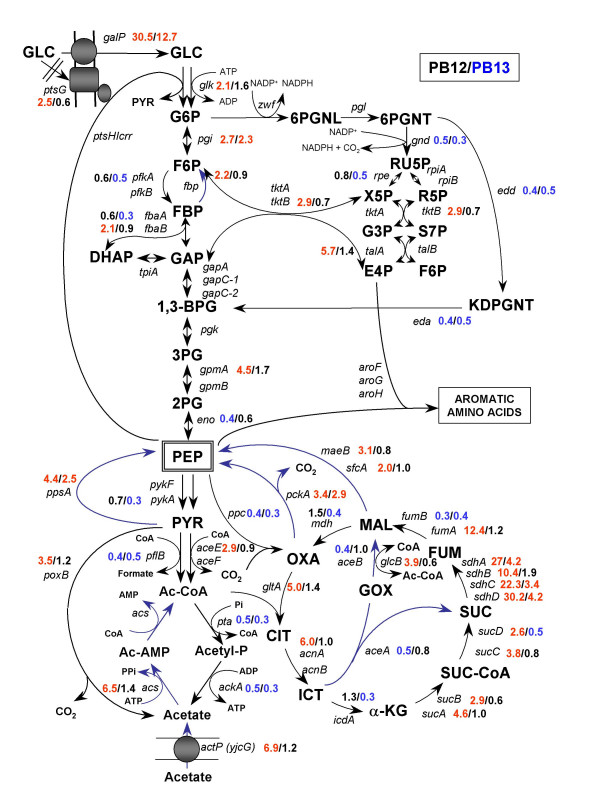
**Relative transcript levels for genes from carbon central metabolism for PB12 (first value) and PB13 (second value), as compared to JM101**. Metabolic network showing the relative gene transcription levels of genes related to the carbon central metabolism, fermentative pathways and the connection with the common aromatic amino acid pathway. Most relevant transcriptional responses in host strains without plasmids are shown: PB12 (first value), PB13 (second value) as compared to JM101. According to the significance criterion, only those relative gene transcription values ≥ 2 (up-regulation, data in red) or ≤ 0.5 (down-regulation, data in blue), as compared to JM101 reference strain, are shown. The relative gene transcription value for JM101 is always equal to 1 and for that reason was omitted. No significant values were written in black. Metabolites abbreviations: GLC, glucose; G6P, glucose-6-phosphate; F6P, fructose-6-phosphate; PBP, fructose-1,6-biphosphate; DHAP, dihydroxyacetone phosphate; GAP, glyceraldehyde 3-phosphate; 1,3-BGP, 1,3-biphosphoglycerate; 3PG, 3-phosphoglycerate; 2PG, 2-phophoglycerate; PEP, phosphoenolpyruvate; PYR, pyruvate; 6PGLN, 6-phosphoglucono-δ-lactone; 6PGNT, 6-phophogluconate; Ru5P, ribulose-5-phosphate; R5P, ribose-5-phosphate; Xu5P, xylulose-5-phosphate; S7P, sedoheptulose-7-phosphate; E4P, erythrose-4-phosphate; Ac-CoA, acetyl coenzyme A; Ac-P, acetyl phosphate; Ac-AMP, acetyl-AMP; CIT, citrate; ICT, isocitrate; GOX, glyoxylate; α-KG, α-ketoglutarate; SUC-CoA, succinyl-coenzyme A, SUC, succinate; FUM, fumarate; MAL, malate; OXA, oxaloacetate.

**Figure 4:**
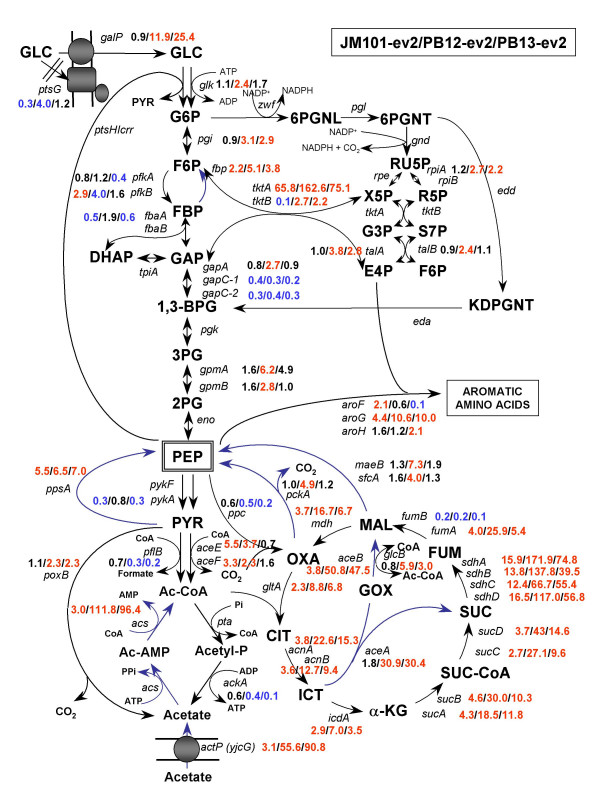
**Relative transcript levels for genes from carbon central metabolism for L-Phe overproducing strains**. Metabolic network showing the relative gene transcription levels of genes related to the carbon central metabolism, fermentative pathways and the connection with the common aromatic amino acid pathway. Most relevant transcriptional responses in L-Phe overproducing strains are shown: JM101-ev2 (first value), PB12-ev2 (second value) and PB13-ev3 (third value), as compared to JM101. Metabolites abbreviations are depicted in Figure 3 legend.

Earlier studies have demonstrated that glucose permeability of the outer membrane in *E. coli *is determined by the major outer membrane porins OmpF, OmpC and LamB [16]. MTA showed no significant changes of *lamB *transcription in PB12 or PB13 mutants, in contrast with PTS mutants transformed with plasmids where it was highly induced in PB12-ev2 (34-fold) and PB13-ev2 (14-fold). No changes were detected in the transcript levels of *ompF *among all the strains (additional file 1). As can be observed, the transcription of *mlgB *and *galP *genes was highly induced in a PTS background, while *lamB *and *malE *transcription was strongly up-regulated by both PTS inactivation and presence of the plasmids. The mechanisms underling these up-regulations remain unknown. However, *mglB *and *galP *genes have in common similar regulatory proteins: cAMP-CRP complex, GalS and GalR (additional file 1). Accordingly, the regulatory mechanism involved in up-regulation of *mglB *may be a consequence of the GalS and GalR inactivation by endogenous galactose synthesis. It can be observed that gene expression patterns of *lamB *bear resemblance to those of *malE *gene. In addition, these genes share some regulatory proteins such as CRP and MalT. Hence it is possible to speculate that the regulatory mechanism of *lamB *and *malE *inductions in PB12-ev2 and PB12-ev2 may be mediated by cAMP-CRP complex and/or MalT.

### Glycolysis, gluconeogenesis and anaplerotic pathways

Figures 3 and 4 show the most relevant transcriptional changes for genes from glycolysis (EMP), pentoses phosphate (PPP), Entner-Doudoroff pathways and TCA cycle. These figures show relative transcript levels in strains PB12, PB13, JM101-ev2, PB12-ev2 and PB13-ev2. The gene encoding glucokinase (*glk*) was slightly up-regulated only in PB12 (2.1-fold) and PB12-ev2 (2.4-fold) (additional file 1, Figures 3, 4). In wild type *E. coli *strains, the glucose transporter of PTS (EIIBC^Glc ^component) couples the translocation with the phosphorylation of glucose. PTS is the usual route for the internalization and glucose phosphorylation, while glucokinase (Glk) plays a minor role in glycolysis. However, when PTS is inactivated, Glk acquires great significance, because inactivation of *glk *in a PTS^- ^background causes inability to grow on glucose [30,31]. *glk *transcription is controlled by the fructose repressor FruR, also known as Cra (Catabolite repressor/activator) [32,33]. In addition, *glk *induction in response to stress caused by the overexpression of foreign proteins [34] and also in response to PTS deletion (PB12 mutant) has been reported [18].

The next glycolytic step is catalyzed by phosphoglucose isomerase (Pgi). Transcription of the *pgi *gene was increased in the PTS^-^Glc^+ ^background: PB12 (2.7-fold), PB13 (2.3-fold), PB12-ev2 (3.1-fold), PB13-ev2 (2.9-fold). Despite the significance of Pgi in glycolysis, scarce information is available about the regulation of the *pgi *gene.

Phosphofructokinase (Pfk) plays a central role in the control of the glycolytic flux because it catalyzes one of the pathway's rate-determining reactions. In *E. coli*, two distinct enzymes perform this reaction, Pfk-1 (*pfkA*) and Pfk-2 (*pfkB*), contributing with 90% and 10% of the total activity, respectively [31]. Therefore, Pfk-1 is the major phosphofructokinase activity in *E. coli*. Figures 3 and 4 show that *pfkA *transcription was slightly down-regulated in PB13 and PB13-ev2, while, the *pfkB *gene was induced in JM101-ev2 (2.9-fold) and PB12-ev2 (4-fold). It is known that the Cra and CsrA/csrB systems are involved in the regulation of the *pfkA *gene [32], but no information is available about *pfkB *regulation.

In general, only slight changes were detected in the transcription level of genes related to glycolysis among the strains. For instance, the *gapA *transcript, encoding G3P dehydrogenase, was only increased in PB12-ev2 (2.7-fold). In addition, *gpmA *transcript, encoding phosphoglycerate mutase 1, was significantly increased in PB12 (4.5-fold), PB12-ev2 (6.2-fold) and PB13-ev2 (4.9-fold), while *gpmB *was only induced in PB12-ev2 (2.8-fold) (additional file 1, Figures 3 and 4). Several glycolytic genes, showed no significant changes in the transcription profiles among all strains. On the other hand, some of these genes showed down-regulation responses, for example in PB12 only the transcript levels of *eno *were down-regulated while in PB13, the transcripts of *pfkA*, *fbaA *and *pykA *showed significant repression. The presence of plasmids caused down-regulation of *fbaA, tpiA, gapC-1, gapC-2 *and *pykA *transcript levels in JM101-ev2, while in PB12-ev2 were only down-regulated the transcripts of *gapC-1 *and *gapC-2*. In PB13-ev2, *pfkA*, *gapC-1*, *gapC-2*, *eno *y *pykA *transcript levels were also down-regulated.

Glycolysis and gluconeogenic pathways share seven of ten enzymatic reactions, which are reversible reactions. Both pathways differ in three reactions that permit that both directions be thermodynamically favourable under different physiological conditions. In *E. coli*, one of these distinct bypasses in the gluconeogenic pathway is performed by the fructose-1, 6-biphosphatase enzyme (*fbp*), which catalyzes the opposite reaction to Pfk, converting fructose 1,6-biphosphate to fructose 6-phosphate when cells are growing in gluconeogenic substrates. Transcription of *fbp *was moderately up-regulated in PB12 (2.2-fold), as well as in JM101-ev2 (2.2-fold), PB12-ev2 (5.1-fold) and PB13-ev3 (3.8-fold) mutants (additional file 1). Another bypass reaction in gluconeogenesis is the conversion of pyruvate to PEP. This opposite reaction to pyruvate kinase activity is carried out by the PEP synthetase (Pps) (Figures 3, 4). The relative transcription level of *ppsA *was significantly up-regulated in response to PTS inactivation and/or plasmidic presence, as can be observed in PB12 (4.4-fold), PB13 (2.5-fold), JM101 (5.5-fold), PB12-ev2 (6.5-fold) and PB13-ev2 (7.0-fold). In addition, some other gluconeogenic genes such as *pckA *(PEP carboxykinase) showed up-regulation in PB12 (3.4-fold), PB13 (2.9-fold) and PB12-ev2 (4.9-fold). Likewise, the transcription of genes encoding malic enzymes, *maeB *and *sfcA *were only up-regulated in PB12 and PB12-ev2 as compared to JM101, while the *sfcA *was 2-fold up-regulated in PB12 and 4-fold in PB12-ev2. Noticeably, gluconeogenic genes such as *fbp*, *ppsA*, *pckA*, *maeB *and *sfcA *exhibited a clear transcriptional response toward up-regulation in PB12 and PB12-ev2. It is known that crsA/csrB system regulates *fbp *and *ppsA *[35-37], while Cra regulates *ppsA *and *pckA *[32,33], but no information is available about *maeB *or *sfcA *genes.

### Dissimilation of pyruvate to organic acids

Pyruvate formate-lyase (PFL) encoded by *pflB*, is a key enzyme in the oxidation of pyruvate to acetyl-CoA and formate when *E. coli *is growing in anaerobic conditions [38]. In the present study, *E. coli *cells were grown exponentially in aerobic conditions and utilized for the Metabolic Expression Analysis. The transcript levels of *pflB *were down-regulated in all PTS mutants (with or without plasmids). Pyruvate dehydrogenase (Ldh) complex, encoded by the *aceEF-lpdA *operon, oxidizes pyruvate to acetyl-CoA and CO_2_. MTA showed that transcript levels of the *aceEF *were significantly up-regulated in JM101-ev2 and PB12-ev2 (additional file 1, Figures 3, 4). By contrast, the mRNA levels of the *pta*-*ackA *operon, encoding the enzymes phosphotranscetylase and acetate kinase, respectively, showed down-regulation in PB12 and PB13, but in PB12-ev2 and PB13-ev2 only the transcripts of *ackA *were significantly down-regulated. In addition, the transcription of *poxB *gene showed up-regulation in PB12 (3.5-fold), PB12-ev2 (2.3-fold) and PB13-ev2 (2.3-fold) (additional file 1, Figures 3, 4).

The *acs *gene encodes an acetyl-CoA synthase (Acs), a high-affinity acetate-scavenging enzyme, which permits *E. coli *to survive during starvation periods, utilizing acetate as a source of energy and building blocks. Thus, acetate can be further metabolized to produce acetyl-CoA during periods of nutrient depletion. MTA showed a moderated up-regulation of the transcription of the *acs-actP *operon in PB12 (~6-fold) and JM101-ev2 (~3-fold), whereas this operon was highly induced in PB12-ev2 (*acs*: 111-fold, *actP*: 55-fold) and PB13-ev2 (*acs*: 96-fold, *actP*: 90-fold). It is known that PTS mutants produce substantially lower amounts of acetate as compared to wild type strains [39]. In a previous report, it was shown that PB12-ev2 and PB13-ev2 mutants in physiological conditions of resting cells, accumulate 57 and 42% less acetate than JM101, respectively (table 1)[15]. In addition, it was recently demonstrated that glucose catabolic repression in PTS mutants growing on glucose and acetate has been abolished, leading to the simultaneous consumption of both carbon sources [18,25,40]. All this information is consistent with the current MFA, showing a good correlation between the down-regulation of genes involved in the production of acetate (*pta-ackA *operon), the up-regulation of genes related to acetate consumption and transport (*acs-actP *operon) and lower acetate accumulation in PTS mutants. On the other hand, the up-regulation of *acs-actP *operon and down-regulation of *pta-ackA *operon may be transcriptional responses to the alteration of PEP or pyruvate pools caused by the PTS inactivation and/or redirection of carbon flux from central metabolism to the SHIK pathway. These results might indicate that the cell senses nutritional stress conditions and trigger transcriptional responses to increases the gluconeogenic capacity, incorporating alternative carbon sources such as acetate or inducing genes related to carbohydrate transport. A similar transcriptional response to nutrimental stress has been previously observed in PB11 mutant grown in minimal media supplemented with glucose [18,25].

### Tricarboxylic Acid Cycle (TCA) and Glyoxylate Bypass

The transcription of *gltA*, encoding the citrate synthase, was up-regulated in strain PB12 (5-fold) and in strains harbouring plasmids: JM101-ev2 (2.3-fold), PB12-ev2 (8.8-fold), and PB13-ev2 (6.8-fold). A similar trend was observed for the same strains with regard to *acnA *transcription levels, encoding aconitase isoenzyme, showing up-regulation in PB12 (6-fold), and JM101-ev2 (3.8-fold), PB12-ev2 (22.6-fold) and PB13-ev2 (15.3-fold). The transcripts levels of *acnB *(aconitase B) and *icdA *(isocitrate dehydrogenase) were only up-regulated in the presence of the plasmids in JM101-ev2 (3.6-fold), PB12-ev2 (12.7-fold) and PB13-ev2 (9.4-fold), but they did not change in PB12, whereas, in contrast, they were slightly down-regulated in PB13 (0.3–0.4 fold). On the other hand, consistently with its operon organization, the transcription of the *sucABCD*, encoding 2-oxoglutate dehydrogenase and succinyl-CoA synthase, wasup-regulated in PB12 (2.6- to 4.6-fold) and JM101-ev2 (2.7–4.6 fold) but not in PB13, while it was further induced by the presence of plasmids in PB12-ev2 (18- to 43-fold) and in PB13-ev2 (9- to 14-fold) (additional file 1). A stronger induction of the *sdhCDAB *operon transcription, encoding succinate dehydrogenase, was detected in PB12 (10- to 30-fold), PB13 (2- to 4-fold) and JM101-ev2 (12- to 16-fold), which was strongly increased in PB12-ev2 (66- to 170-fold), PB13-ev2 (39- to 74-fold). Some genes such as *fumA *and *fumB*, encoding two fumarase isoenzymes, were differentially regulated among strains. Thus, *fumA *transcription was up-regulated in PB12 (12.4-fold), JM101-ev2 (4-fold), PB13-ev2 (5.4-fold) and strongly induced in PB12-ev2 (25.9-fold). On the contrary, *fumB *transcription was down-regulated in all strains. The transcription of *mdh *showed a pattern similar to *acnB *and *icdA*, which was up-regulated in the presence of the plasmids in JM101-ev2 (3.5-fold), PB12-ev2 (16.7-fold) and PB13-ev2 (6.7-fold), but it did not change in PB12, whereas it was slightly down-regulated in PB13 (additional file 1). With regard to the glyoxylate shunt pathway, the transcription of *aceBA *operon, encoding malate synthase A and isocitrate lyase, respectively, was strongly up-regulated only in PB12-ev2 (*aceA*: 30-fold, *aceB*: 50-fold) and PB13-ev2 (*aceA*: 30-fold, *aceB*: 47-fold). Alternatively, the transcripts of *glcB *(malate synthase G) were measured, showing up-regulation in PB12 (3.9-fold), PB12-ev2 (5.9-fold) and PB13-ev2 (3-fold).

Analyzing the current results, it is evident that transcriptional profiles in most of TCA and glyoxylate genes show a general trend toward up-regulation in PB12, JM101-ev2, PB12-ev2 and PB13-ev2 (additional file 1). In contrast, PB13 mutant only showed up-regulation of the *sdhCDAB *operon, while the rest of TCA genes remained without significant changes. It is important to emphasize that a mutation in *arcB *gene of PB12 strain has been detected, which consists in a substitution of Tyr by Cys residue at position 71 and this mutation is not present in PB13. It can be assumed that t6he *arcB *mutation is responsible for the slight up-regulation of most of TCA cycle genes in PB12 strain when growing on glucose as the sole carbon source [18]. Possibly, it may also explain the differences in the transcription patterns of most of the TCA cycle genes observed between PB12 and PB13 strains. However, the presence of plasmids in JM101-ev2, PB12-ev2 and PB13-ev2 introduces an additional factor, which up-regulated most of TCA cycle genes in all these strains by a mechanism still unknown. It should be noted that transcript levels of TCA cycle genes is higher in PB12-ev2 than in JM101-ev2 and PB13-ev2.

### Genes encoding regulatory proteins of carbon central metabolism

With the purpose of finding some correlations between transcriptional changes of metabolic genes with variations in the transcript levels of genes coding regulatory proteins of metabolic genes, we measured transcript levels of some regulatory proteins such as Cra, RpoD, RpoS, FNR, CsrA/crsB and ArcA/ArcB systems [32-51]. Despite transcript levels of some regulatory genes such as *rpoS*, *csrB*, *rpoD*, *arcA *and *fnr *were significantly regulated in some strains (additional file 1), however, no clear correlation was found between gene expression patterns of regulatory genes with those of the genes regulated by them. In order to establish correlations in these gene expression patterns, it would be necessary to know the phosphorylation state of the regulatory protein or the presence/concentration of specific cofactors that alter their activity.

### Pentoses phosphate and Entner-Doudoroff pathway

The *zwf *gene encodes G6P dehydrogenase (G6PDH) and its regulation is growth-rate dependent [52]. Like *poxB *gene, *zwf *is activated by SoxS in response to oxidative stress [53] and by the regulator of multiple resistance antibiotics, Mar [54]. The G6PDH activity plays an important role in controlling carbon distribution at the G6P node, because it directs carbon flux through the oxidative branch of the pentose phosphate pathway (PPP) depending on NADP^+ ^availability. MTA showed that relative transcription of *zwf *was down-regulated in PB13-ev2, while the transcription of *gnd *gene, encoding the phosphogluconate dehydrogenase was down-regulated in PB12 and PB13 (figure 3). Isomerization and epimerization reactions of ribulose-5-P interconnect the oxidative and non-oxidative branches of PPP and are coded by *rpe *and *rpiA *genes, respectively. The transcription of *rpe *gene was down-regulated only in PB13, while the *rpiA *transcription was slightly up-regulated in PB12-ev2 (2.7-fold) and PB13-ev2 (2.2-fold). Enzymes constituting the non-oxidative branch of PPP, such as transketolases and transaldolases activities interconnect glycolysis with the oxidative branch of PPP. In *E. coli*, each of these activities is catalyzed by two isoenzymes, encoded by *tktA*, *tktB *and *talA*, *talB *for transketolase and transaldolase activities, respectively. Transketolase A (*tktA*) is the major isoenzyme and accounts for about 70–80% of transketolase activity [55,56]. As expected, the overexpression of *tktA *gene from the plasmid pCLtkt strongly increased the transcripts levels of *tktA *in JM101-ev2 (65.8-fold), PB12-ev2 (162.6-fold) and PB13-ev2 (75.1-fold), as compared to chromosomal *tktA *transcription in JM101. It is worth to emphasize the difference in the levels of *tktA *transcription among these strains, which varied depending on the genetic background, reaching its maximum value in PB12-ev2. The regulatory mechanism implicated in the variation of *tktA *transcript levels is unknown. This transcriptional variability can be of significance and should be taken into account, especially when engineering distinct mutants by using multicopy plasmids. In the context of aromatic amino acid production, the transketolase overexpression improves significantly the yield of aromatic compounds, presumably by increasing the availability of E4P [6]. Therefore, the different transcript levels of *tktA *detected among the strains may affect the yield and productivity of L-Phe. The *tktA *gene, either the chromosomal or plasmid copy, is under the control of its own regulatory region.

Transcription of the *talA*-*tktB *operon exhibited similar patterns, which is consistent with their transcriptional organization. Thus, the relative gene transcription of *talA *was up-regulated in PB12, PB12-ev2 and PB13-ev2 by 5.7-, 3.8- and 2.8-fold, respectively, while the transcripts of *tktB *were also induced in PB12, PB12-ev2, PB13-ev2 by 2.9-, 2.7- and 2.2-fold, respectively. It has been demonstrated that overexpression of either transketolase or transaldolase has a positive effect on the synthesis of aromatic compounds. For this reason, the significant induction of *talA-tktB *operon in PB12-ev2 PB13-ev2 results of particular interest for L-Phe production. It is known that the CreBC two-component system positively regulates the transcription of the *talA*-*tktB *operon in *E. coli *cells growing in minimal media [57], but it is not known which is the signal or the regulatory mechanism responsible of this induction.

The *edd-eda *operon encodes enzymes of the Entner-Doudoroff pathway (EDP)[58]. No significant changes were observed in the transcription levels of *edd *and *eda *genes among all strains (additional file 1).

### Common and specific pathways of aromatic amino acids

The common aromatic amino acid pathway, so-called shikimate (SHIK) pathway, encompasses seven serial enzymatic reactions. The first reaction is catalyzed by the enzyme DAHP synthase, which condensates E4P and PEP to yield DAHP. *E. coli *has three DAHP synthases isoenzymes encoded by *aroF*, *aroG *and *aroH *genes, which are transcriptionally regulated by either transcriptional repressors (TyrR or TrpR) or attenuation mechanisms (additional file 1). As expected, the transcript levels of *aroG*^fbr^, expressed from the plasmid pJLB*aroG*^fbr ^under the control of *lacUV5 *promoter, were higher in JM101-ev2 (4.4-fold), PB12-ev2 (10.6-fold) and PB13-ev2 (10-fold) with regard to the chromosomal level of wild type *aroG *in JM101. However, as can be observed, these levels were significantly different between PTS^+ ^and PTS^-^Glc^+ ^strains for unknown reasons. It is important to note that 10-fold increase in *aroG*^fbr ^transcript levels were apparently enough to overproduce L-Phe at high yields and productivities in PB12-ev2 (table 1). Alternatively, transcript levels of *aroF *and *aroH *were slightly up-regulated (~2-fold) in JM101-ev2 and PB13-ev2, respectively. Transcription control of *aroF *is mediated by the TyrR repressor, whereas *aroH *transcription is repressed by TrpR [59]. Transcriptional analysis showed a slight increase of the *tyrR *mRNAs only in PB12 and PB12-ev2, while up-regulation of *trpR *transcripts were also detected in all strains harbouring plasmids with regard to JM101 (additional file 1). It is known that the repression exerted by TyrR or TrpR is cofactor-dependent (tyrosine or tryptophan)[59]. No apparent correlation was found between the up-regulation of these repressors and the transcripts levels regulated by them, such as *aroF *or *aroH*.

Some genes known to be involved in the synthesis of aromatic amino acids showed a decrease of transcript levels in PB12 and PB13, such as *aroD *(3-dehydroquinate dehydratase) and *aroA *(5-enol-pyruvyl-shikimate 3-phosphate synthase) (additional file 1). MTA revealed that the best L-Phe producing strains (JM101-ev2 and PB12-ev2) showed no down-regulation of genes involved in shikimate pathway (additional file 1); while transcript levels of *aroB *(3-dehydroquinate synthase) were slightly down-regulated (2-fold) in PB13 and PB13-ev2 strains. On the contrary, JM101-ev2 and PB12-ev2 strains showed up-regulation of transcript levels of *aroC *(chorismate synthase) (2–3 fold). Down-regulation of genes may be related to a decreased in enzyme pools and thereby with enzymatic limitation within a pathway. In addition, L-Phe overproducing PB13-ev2 strain also showed down-regulation of the transcript levels of *aroE *(shikimate dehydrogenase) (additional file 1). Therefore, the down-regulation of *aroB*, *aroE *y *aroA *in PB13-ev2 may be associated with the lower yields and specific productivity of L-Phe synthesized from glucose, as compared to either JM101-ev2 or PB12-ev2 (additional file 1). It is important to note that enzymatic levels of shikimate dehydrogenases (*aroE*, *ydiB*) and shikimate kinases (*aroK *and *aroL*) are rate-limiting steps in all L-Phe overproducing strains, as can be inferred by the accumulation of DHS and SHIK (table 1).

### Tryptophan, tyrosine and phenylalanine biosynthetic pathways

The specific biosynthetic pathways of aromatic amino acids are also strongly controlled at the transcriptional and enzymatic level [59]. The biosynthetic pathway of tryptophan (L-Trp) starts with the conversion of CHO to antranilate, catalyzed by the antranilate synthase (coded by *trpE*) and ends with the synthesis of tryptophan. The last step is catalyzed by tryptophan synthase (coded by *trpAB*). The transcript levels of the *trpE *and *trpA *genes belonging to the *trpLEDCBA *operon were measured, showing down-regulation of these genes in all strains, compared to JM101 (additional file 1). *E. coli *utilizes two distinct mechanisms for regulating transcription of the *trpLEDCBA *operon: repression/activation and attenuation [59,60]. Repression of this operon is mediated by TrpR protein. The TrpR regulation mechanism is cofactor-dependent. Likewise, the attenuation of *trp *operon depends on charged tRNA^Trp ^[60,61]. As previously mentioned, the transcript levels of *trpR *were up-regulated about 2–3 fold in strains carrying out plasmids. A positive correlation can be observed between the increase of the transcript levels of repressor *trpR *and the repression of the *trpLEDCBA *operon. However, the presence of tryptophan as cofactor must be considered in this regulation mechanism.

**Figure 5:**
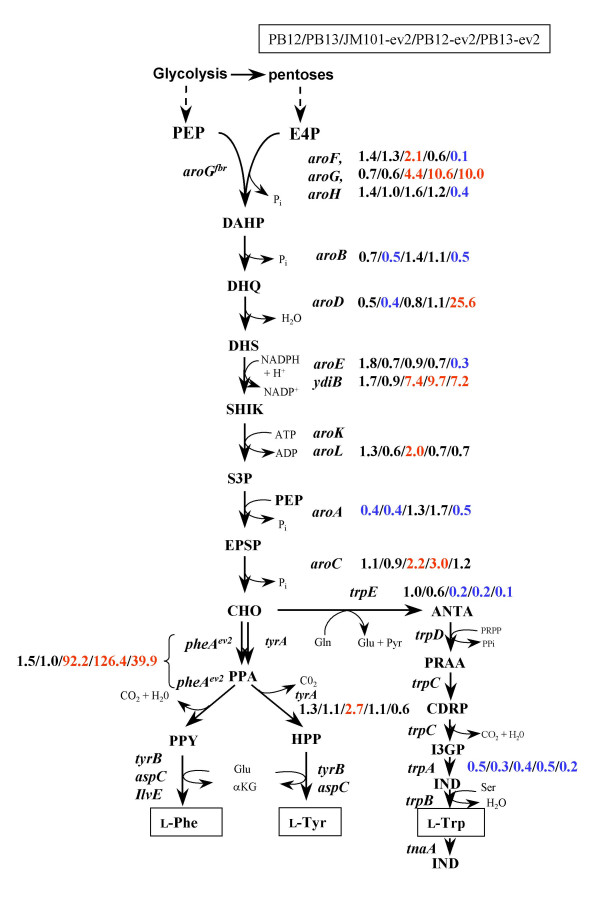
**Relative transcript levels for genes from the shikimate and aromatic specific pathways**. Metabolic transcription analysis of genes related to the shikimate pathway and specific aromatic amino acid pathways. Metabolites abbreviations: PEP, phosphoenolpyruvate; E4P, erythrose 4-phosphate; DAHP, 3-deoxy-D-*arabino*-heptulosonate-7-phosphate; DHQ, 5-dehydroquinate; DHS, 5-dehydroshikimate; SHIK, shikimate; S3P, shikimate 5-phosphate; ESPS, 3-enolpyruvylshimate-5-phosphate; CHO, chorismate; PPA, prephenate; PPY, phenylpyruvate; HPP, 4-hydroxyphenylpyruvate; ANTA, anthranilate; PRAA, N-(5'-Phosphoribosyl)-anthranilate; CDRP, enol-1-o-carboxyphenylamino-1-deoxy-ribulose phosphate; I3GP, indol-3-glycerol phosphate; IND, indole; L-Phe, phenylalanine; L-Tyr, tyrosine; L-Trp, tryptophan, L-Ser, serine; L-Gln, glutamine; L-Glu, glutamate.

The committed step toward the biosynthesis of tyrosine is catalyzed by the bifunctional enzyme chorismate mutase-prephenate dehydrogenase, coded by *tyrA *gene (Figure 5). The mRNA levels of *tyrA *showed a slight up-regulation only in PB12-ev2. The transcription pattern of *tyrA *is similar to that of *aroF *gene (additional file 1), which is consistent with the organization of these genes in the *aroF-tyrA *operon that controlled by TyrR [59,62,63].

In *E. coli*, CHO is converted into prephenate (PPA) by the enzyme chorismate mutase (CM). PPA is then further metabolized by the enzyme prephenate dehydratase (PDT) into phenylpyruvate (PPY); both activities are co-localized in the bifunctional enzyme CM-PDT encoded by *pheA *gene. Both activities of this enzyme are feedback inhibited by L-Phe. It is worth mentioning that for the metabolic transcription analysis of chromosomal *pheA *(wt) and the truncated version of *pheA*^*ev2 *^gene, specific primers were designed in order to distinguish between the mRNA coming from either *pheA *(wt) or *pheA*^*ev2 *^gene (table 3). The specific primers for the PCR amplification of the *pheA *(wt) hybridize in the regulatory domain R, which is absent in *pheA*^*ev2 *^gene. MTA showed that mRNAs levels of chromosomal *pheA *gene were slightly increased only in PB12 (2-fold) but down-regulated in JM101-ev2, PB12-ev2. It is known that transcription of wt *pheA *gene is solely regulated by attenuation at a transcription terminator located upstream from the *pheA *structural gene. According to the established attenuation mechanisms, the amount of charged tRNA^Phe ^(*pheR*) regulates the *pheA *transcription via attenuation control of transcription [59,64,65]. Therefore, altered regulation of wt *pheA *gene may be explained by the mechanism of attenuation. The overexpression of the *pheA*^*ev2 *^transcript from the plasmid pTrc*pheA*^*ev2 *^was clearly evidenced by RT-PCR analysis, showing a very strong up-regulation in JM101-ev2 (92-fold), PB12-ev2 (126-fold) and PB13-ev2 (40-fold). The expression *pheA*^*ev2 *^from the multicopy plasmid also seems to be dependent on the genetic background, having the highest transcription in PB12-ev2. The *pheA*^*ev2 *^gene is under the control of the *trc *promoter, which is inducible by IPTG. Because of the overexpression of *pheA*^*ev2 *^from plasmid pTrc*pheA*^*ev2 *^in the strains JM101-ev2, PB12-ev2 and PB13-ev2, it is assumed that this plasmid is the major source of *pheA *transcripts.

**Table 3:** Nucleotide sequences of the primers sets used for RT-PCR assays. The rest of the primer sets has been already reported [18].

Gene	Primer	Primer sequence
Aromatic amino acid pathways		

*aroF*	aroFa	5' CAGAATCCGTGGTTGCTCAA 3'
	aroFb	5' CGGTTGCTCGGAAGACTGAT 3'
*aroG*	aroGa	5' GTCGGCTTCAAAAATGGCAC 3'
	aroGb	5' CCCCATTTCGTTACGGACAG 3'
*aroH*	aroHa	5' CTGCTCGATCCACGATCTCA 3'
	aroHb	5' AGGTGCGCATTACGATTTCC 3'
*aroB*	aroBa	5' GTTATCCTCCCTGACGGCG 3'
	aroBb	5' ACCAGCGTAGTATCGCGACC 3'
*aroD*	aroDa	5' AGCCGAAGAAATCATTGCCC 3'
	aroDb	5' ACGTCAGCACATCGCTGGTA 3'
*aroE*	aroEa	5' AATCCGATAGCCCACAGCAA 3'
	aroEb	5' TCATTGATGGGTGCCAACAC 3'
*ydiB*	ydiBa	5' AAAAGCGGGATTGCCATTTA 3'
	ydiBb	5' CAGTTCCGCGCATTTTGAG 3'
*aroL*	aroLa	5' CCGGAAGAAGATTTACGGCC 3'
	aroLb	5' TGCGCAACTTCGCGATATAG 3'
*aroK*	aroKa	5' GCACTATTGGGCGCCAGTTA 3'
	aroKb	5' GAAAACCCAGCCCACATCAG 3'
*aroM*	aroMa	5' CCAGAAGCAGGCGAAGACAC 3'
	aroMb	5' TCAACCACACCTTGCAGGTC 3'
*aroA*	aroAa	5' CATATTCCTGATGCGGCGAT 3'
	aroAb	5' TCGGTCTCTTTAACACGCCAG 3'
*aroC*	aroCa	5' GGGACATCGCGCTATACCAC 3'
	aroCb	5' AACAAGCCAATGCTGGTGC 3'
*pheA*	pheARa	5' ATGACCCGTCTGGAATCACG 3'
	pheARb	5' GCTTTTTGCATTTCCGCTG 3'
*pheA-ev2*	pheAa	5' ACTCAGCAGGCTTTGCTCCA 3'
	pheAb	5' GCCGCAAGATGGGAATAAGAA 3'
*tyrA*	tyrAa	5' GTCAGATGGGACGCCTGTTC 3'
	tyrAb	5' GGCAACAATATCAGCCGCTC 3'
*aspC*	aspCa	5' GGCGATTCGCGCTAACTACT 3'
	aspCb	5' GCTCTTGTTCCCAAATCGCA 3'
*trpE*	trpEa	5' CGCAACTGTCAGCGGAAAAT 3'
	trpEb	5' GGCCTGAATACGGGTGCTTT 3'
*trpA*	trpAa	5' CGTTCCTTTCGTCACGCTC 3'
	trpAb	5' CGTTCCTTTCGTCACGCTC 3'

Aromatic amino acid transport		

*pheP*	phePa	5' CGGCTGTATTGCTGGGCTAC 3'
	phePb	5' AAATGAACCGGATACCGGCT 3'
*aroP*	aroPa	5' TGGTAATCAACTGGGCGATG 3'
	aroPb	5' CGGATAAAGCAGAGCAGGGA 3'
*tyrP*	tyrPa	5' CCGGTGTTGGTTTTAGCGTT 3'
	tyrPb	5' CGGAACATGCTGGTACACCTC 3'
*mtr*	mtra	5' TTGATGTGCTGGTACAGGCG 3'
	mtrb	5' GAGGAACGAACTCGCTACCG 3'
*shiA*	shiAb	5' ATTATCAAGCTGCCGCGAAA 3'
	shiAb	5' CAATTCGCACAGTCGTAGCG 3'
*ilvE*	ilvEa	5' AAGGCATCCGTTGCTACGAC 3'
	ilvEb	5' CGGGAAGCGATAGATTTTGG 3'
*aroP*	aroPa	5' TGGTAATCAACTGGGCGATG 3'
	aroPb	5' CGGATAAAGCAGAGCAGGGA 3'

Regulators		

*trpR*	trpRa	5' CAGAACAGCGTCACCAGGAG 3'
	trpRb	5' TGGCGTCAGCATCAGGTTTA 3'
*tyrR*	tyrRa	5' GGCGAGCTGTCAGCTTTTTG 3'
	tyrRb	5' CGCTTTCCAGCCAACGTAAA 3'
*csrA*	csrAa	5' TCTGACTCGTCGAGTTGGTGA 3'
	csrAb	5' GGGCATTTACGCCAATACGT 3'
*rpoD*	rpoDa	5' GATTCTGCGACCACCGAAAG 3'
	rpoDb	5' TCGATACCGAAACGCATACG 3'
*rpoE*	rpoEa	5' GAACTATTGAGTCCCTCCCGG 3'
	rpoEb	5' CGGACAATCCATGATAGCGG 3'
*fnr*	fnra	5' CGGAAAAGCGAATTATACGGC 3'
	fnrb	5' TTCGTTGAGTGTGAACGGGA 3'

Carbohydrate transport		

*malE*	malEa	5' AGAAATTCCCACAGGTTGCG 3'
	malEb	5' TTCAGCCAACAGGCCAGATT 3'

The last step in the biosynthesis of Phe and Tyr requires transamination of HPP or PPY, respectively, with glutamate as amino donor. Although, in *E. coli *there are three enzymes with transaminase activity encoded by *tyrR*, *aspC *and *ilvE *genes, it is seems that under normal physiological conditions Phe and Tyr syntheses are primarily carried out by the product of *tyrB *gene, the aromatic aminotransferase [59]. However, MTA showed no higher levels for the *tyrB *transcripts, while transcript levels of aminotransferase encoded by *ilvE *showed no significant changes among all strains, and *aspC *showed down-regulation in PB12 and PB13.

### Aromatic amino acids transport

The aromatic amino acids are imported across the inner membrane of *E. coli *by distinct transport systems. The *aroP *gene encodes the general aromatic transport system for all three aromatic amino acids, while *pheP*, *tyrP *and *mtr *genes encode specific transporters of high-affinity for L-Phe, L-Tyr and L-Trp, respectively. L-Trp is also transported by the low-affinity transporter encoded by *tnaB*, a member of the *tnaAB *operon. It is not known whether these transporters are capable to excrete the aromatic amino acids as well. MTA detected variations in the transcriptional expression of genes related to aromatic transport, showing that mRNA levels of *aroP *(Phe/Tyr/Trp transporter)*pheP *(Phetransporter) and *tyrP *(Tyr transporter)were up-regulated in JM101-ev2 (2- to 5-fold). Similarly, *pheP *and *mtr *(*Trp *transporter) transcript levels showed up-regulation in PB12-ev2 (2- to 3-fold) (additional file 1). The transcript levels of the *shiA *gene (shikimate transporter) were slightly down-regulated in PB12, JM101-ev2, PB12-ev2, and PB13-ev2 (additional file 1).

### Reverse engineering strategies to further improve the L-Phe biosynthesis

One approach considers the analysis of transcriptional responses in the best overproducing strains (PB12-ev2), which could be responsible for the improvement of biosynthetic capacities. This analysis can provide the basis for the implementation of appropriate genetic strategies in order to modulate the transcription of target genes and achieve similar transcriptional responses. For instance, one of the most outstanding transcriptional responses triggered by PTS inactivation and the presence of plasmids (PB12-ev2, PB13-ev2) was the up-regulation of the *acs-actP *operon. This response suggests that PTS mutants have increased their capacity to uptake and metabolize acetate, which is in agreement with lower accumulation of acetate in these mutants. Therefore, the overexpression of *acs-actP *operon may be useful to reincorporate by-products, such as acetate, into the central metabolism, and then, to channel them into the desired production pathway. In fact, the overexpression of Acs in *E. coli *has been recently assessed, resulting in a significant decrease of acetate accumulation and more efficient acetate assimilation [66]. However, the overexpression of ActP has not been assessed yet. We propose that simultaneous gene overexpression of these two genes may have a synergistic effect, increasing the cell capacity to reincorporate acetate into central metabolism, and thereby, decreasing the carbon waste as byproducts and the acetate toxicity in the microbial cultures as well.

MTA showed a differential regulation of some genes that are exclusively gluconeogenic even between mutants with the same PTS^-^Glc^+ ^phenotype. Thus, *fbp, ppsA, pckA, maeB, sfcA *and glyoxylate shunt genes showed a coordinated up-regulation in PB12 and PB12-ev2 strains. Unlike, PB13, PB13-ev2, and JM101-ev2 strains showed only up-regulation of two gluconeogenic genes (*fbp*, *ppsA or pckA*). It has been reported that the overexpression of the *fbp *gene significantly improved lysine production in *C. glutamicum *[67]. This improvement was associated to the increase of carbon flux through oxidative branch of the PP pathway and the concomitant increase in the production NADPH, which is required in the biosynthesis of lysine. In L-Phe biosynthetic pathway, NADPH is also required; therefore, it is possible to propose that coordinated expression of some exclusively gluconeogenic genes may have a positive impact to improve the biosynthesis of aromatic compounds.

## Conclusion

• Many genes exhibited opposite transcriptional trends towards either down- or up-regulation, as a consequence of the PTS inactivation.

• The presence of multicopy plasmids caused a clear transcriptional trend mainly toward up-regulation of a broad spectrum of genes, and not toward down-regulation. The functions of the genes involved in the observed response suggest an increased demand for energy and/or precursor molecules synthesis, which in turn, is an indication of plasmid burden.

• The most outstanding differences in transcriptional responses of metabolic genes between PB12 and PB13 were found in genes related to TCA cycle and glyoxylate shunt.

• Transcription levels of a gene expressed from the same plasmid (as *tktA *and *pheA*^ev2^) was found to strongly vary depending on the genetic background of the host strains. This factor may affect yields and productivities in engineered strains.

• Only few transcriptional changes were detected in the shikimate pathway. Two genes, *aroD *and *aroA*, were down-regulated in PB12 and PB13 as consequence of PTS inactivation. In the presence of plasmids, only PB13-ev2 showed down-regulation of *aroB*, *aroE *and *aroA *genes. Some genes involved in the aromatic amino acid transport were significantly induced in JM101-ev2 (*aroP*, *pheP*, *tyrP*) and PB12-ev2 (*pheP*, *mtr*).

• Noticeably, some exclusive gluconeogenic genes such as *fbp, ppsA, pckA, maeB, sfcA *and glyoxylate shunt genes showed a simultaneous up-regulation in PB12 and PB12-ev2 strains. Unlike, PB13, PB13-ev2, and JM101-ev2 strains showed only up-regulation of two gluconeogenic genes (*fbp*, *ppsA or pckA*).

• Some genes encoding regulatory proteins of metabolic genes showed significant changes in transcript level on some of the different genetic backgrounds studied. However, because the activity of most regulatory proteins is dependent on either cofactor binding or phosphorylation state, and this information was not known, it was not possible to find a clear correlation between transcriptional levels of genes encoding regulatory proteins and their corresponding regulated genes.

## Methods

### Strains, plasmids and construction of L-Phe overproducing strains

All strains and plasmids used in this study are listed and described in table I. *Escherichia coli *JM101 (PTS^+^) is the parental strain of PB11, PB12 and PB13 mutants. Details about the construction of the plasmids pJLB*aroG*^fbr^, pCL*tkt*, pTrc*pheA*^ev2^, as well as the generation of the evolved feedback insensitive chorismate-mutase prefenate-dehydratase, CM-PDT^ev2 ^(*pheA*^ev2^), have been described [15]. For the generation of L-Phe overproducing mutants, JM101, PB12 and PB13 strains were transformed with three plasmids harbouring genes that encode for a feedback inhibition resistant DAHP synthase (pJLB*aroG*^fbr^), transketolase (pCL*tkt*) and truncated evolved CM-PDT^ev2 ^(pTrc*pheA*^ev2^)(table I). The L-Phe overproducing mutants were designated JM101-ev2, PB12-ev2 and PB13-ev2.

### Growth media and culture conditions

All stock cultures were stored at -70°C in Luria-Bertani medium containing 50% of glycerol. Overnight cultures were prepared in M9 medium supplemented with 5 g/l of yeast extract and 10 g/l of glucose. Then, the cells were subcultured by duplicate in shake flasks containing 50 ml of the same media mentioned above at 37°C and 250 r.p.m. In the case of transformed strains, appropriate antibiotics were added for plasmid maintenance and 100 μM of IPTG for induction of the *aroG*^fbr ^and *pheA*^ev2 ^genes. Neither antibiotics nor IPTG were added for strains without plasmids. Cells were harvested by centrifugation when growing in exponential phase (OD_600nm_= 2) for RNA purification. The culture procedure to prepare the cells for transcriptome analyses was exactly the same reported previously to prepare the resting cells for L-Phe production cultures [15]. This analogy was chosen to correlate the transcriptome analysis with the L-Phe production cultures data.

### RNA extraction and cDNA synthesis

Total RNA extraction was performed using hot phenol equilibrated with water [40]. After extractions, RNA was precipitated with 3 M sodium acetate/ethanol and centrifuged 20,000 g, 15 min., at 4°C. Supernatant was discarded and the RNA suspended in water. RNA was treated with DNAse kit (DNA-free, Ambion) and its concentration carefully measured by densitometry in agarose gels and by 260/280 nm ratio absorbance. cDNA was synthesized using RevertAid H First Strand cDNA Synthesis kit (Fermentas Inc.) and a mixture of specific DNA primers b (Table 2). cDNA was used as template for RT-PCR assays. Reproducibility of this procedure was determined by performing two separate cDNA synthesis experiments from the RNA extracted for each strain. Similar results were obtained for the transcription levels of all these duplicate experiments.

**Table 2:** *E. coli *strains and plasmids used for the construction of L-phenylalanine overproducing strains in this study.

**Strains**	**Relevant features**	**Reference number**
**JM101**	*supE*, *thi*, Δ(*lac-proAB*), F'	70
**PB11**	Derived from JM101, but Δ*ptsHI-crr*; impaired growth on glucose as sole carbon source.	2
**PB12**	Derived from PB11; PB12 grows faster than PB11 on glucose	2
**PB13**	Derived from PB11; PB13 grows faster than PB11 on glucose.	2

**Plasmids**		

**pCL*tkt***	*tktA *(comes from replicon pCL1920, resistant to streptomycin or spectinomycin.	6
**pJLB*aroG*^*fbr*^**	*aroG*^*fbr *^under the control of *lacUV5 *promoter, *lacI*^q ^and *tet *genes. Replication origin from pACYC184.	15
**pTrc*pheA*^*ev2*^**	Evolved feedback insensitive *pheA*^*ev2 *^under the control of lacUV5 promoter. Ev2 superscript means 2^nd ^version of evolved *pheA*^*fbr *^gene.	15

**Engineered strains**		

**JM101-ev2**	JM101 transformed with pJLB*aroG*^*fbr*^, pCL*tkt *and pTrc*pheA*^*ev2*^	15
**PB12-ev2**	PB12 transformed with pJLB*aroG*^*fbr*^, pCL*tkt *and pTrc*pheA*^*ev2*^	15
**PB13-ev2**	PB13 transformed with pJLB*aroG*^*fbr*^, pCL*tkt *and pTrc*pheA*^*ev2*^	69

### Real-time PCR

**Figure 6:**
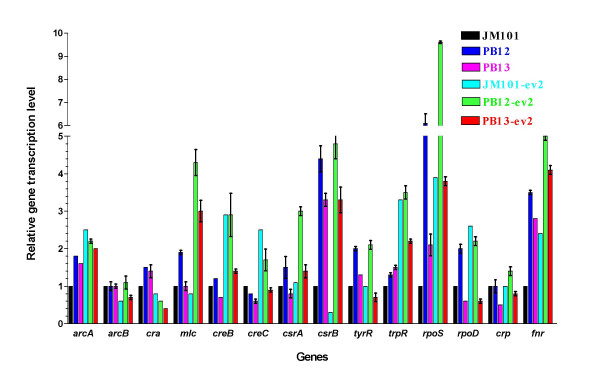
**Relative transcript levels for genes encoding regulatory proteins**. Gene transcription profiling of genes encoding regulatory proteins of central metabolism and aromatic amino acid pathways are shown.

Real-time PCR (RT-PCR) was performed with the ABI Prism7000 Sequence Detection System (Perkin-Elmer/Applied Biosystems) using the SYBR Green PCR Master Mix (Perkin-Elmer/Applied Biosystems). Amplification conditions were 10 min at 95°C, and a two step cycle at 95°C for 15 s and 60°C for 60 s for a total of 40 cycles. The primers for specific amplification were designed using the Primer Express software (PE Applied Biosystems). Most primers used for gene amplification of glycolysis, gluconeogenesis, anaplerosis, PPP, TCA, Entner-Doudoroff, glucose transport have been already reported [18,40]. Table 3 shows the sequences of primers sets used for amplification of genes related to the metabolism of aromatic amino acids (pathways, transport and global regulators). The size of all amplimers was 101 bp. The final primer mix concentration (a plus b) of a total volume of 15 μl was 0.2 μM. 5 nanograms of target cDNA for each gene was added to the reaction mixture. All experiments were performed in triplicate for each gene of each strain, obtaining very similar values (differences of less than 0.3 SD). A non-template control reaction mixture was included for each gene. The quantification technique used to analyze data was the 2^-ΔΔCT ^method described by Livak and Shmittgen [68] and the results were plotted. The data were normalized using the *ihfB *gene as an internal control (housekeeping gene). We detected the same transcription level of this gene in all the strains in the conditions in which the bacteria were grown. JM101 was used as reference strain for all strains, either strains with or without plasmids. The transcript levels of PB12, PB13, PB12-ev2 and PB13-ev2 were normalized subtracting first the housekeeping gene value, and then subtracting the corresponding transcript value of reference strain (JM101) of the same gene. Thus, all the transcripts levels of JM101 were arbitrarily adjusted to one. Therefore, the gene transcription data plotted in all Figures are expressed as relative transcription to JM101 strain. It is important to comment that relative gene transcription in table 3 only allows the comparison of transcript levels of the same gene among all strains, but not among distinct genes. Results of relative transcription showed in additional file 1 and Figures 2, 3, 4, 5, 6 are the averages of six measurements of the RT-PCR transcription values for each gene. Half of the values were obtained from two different cDNAs generated in independent experiments. The RT-PCR transcription values obtained for each gene differ less than 30%.

## Authors' contributions

JLBV participated in the design of this study, performance of cloning, construction of the L-Phe overproducing strains, performed RT-PCR experiments, data analysis and wrote the manuscript. NF participated in the design of oligonucleotides, performed RT-PCR experiments and data analysis. PCE and KJ reviewed and commented the manuscript. GGL and FB participated in results analysis, writing and critical review of the manuscript. All authors have read and approved the manuscript.

## Supplementary Material

Additional file 1 Relative transcript levels determined by RT-PCR in JM101, PB12, PB13 and their corresponding L-Phe overproducing strains JM101-ev2, PB12-ev2 and PB13-ev2 strains. Most of the transcriptional regulation data were taken from EcoCyc database [71]. Relative gene transcription values ≥ 2 are in red (up-regulation), values ≤ 0.5 are in blue (down-regulation). No significant values are in black. Click here for file
