# Supplementary material for: Metabolic transcription analysis of engineered Escherichia coli strains that overproduce L-phenylalanine
Source: Microb Cell Fact. 2007 Sep 19;6:30. doi: 10.1186/1475-2859-6-30 (PMC2089068; doi:10.1186/1475-2859-6-30)
Supplement: Additional file 1 — Relative transcript levels determined by RT-PCR in JM101, PB12, PB13 and their corresponding L-Phe overproducing strains JM101-ev2, PB12-ev2 and PB13-ev2 strains. Most of the transcriptional regulation data were taken from EcoCyc database [71]. Relative gene transcription values ≥ 2 are in red (up-regulation), values ≤ 0.5 are in blue (down-regulation). No significant values are in black. [file 1475-2859-6-30-S1.doc]

|  | |  | | | | **Relative transcript levels as 2^–ΔΔCt^ with JM101 as normalizing value** | | | | | | | | | | | | | | |  |  | | | | | | | |  | | | | | |
| --- | --- | --- | --- | --- | --- | --- | --- | --- | --- | --- | --- | --- | --- | --- | --- | --- | --- | --- | --- | --- | --- | --- | --- | --- | --- | --- | --- | --- | --- | --- | --- | --- | --- | --- | --- |
| **Gene** | | | **Gene product name** | | **PB12** | | **PB13** | | **JM101**  **-ev2** | | | | **PB12**  **-ev2** | | | **PB13**  **-ev2** | | | | | | | **Known regulator** | | | | | | | | | | |  | |
|  | **Glucose transport** | | | | | | | | | | | | | | | | | | | | | | | | | | | |  | | | | | | |
| ***ptsG*** | | | PTS enzyme IIB and IIC domains | 2.5 ± 0.2 | | | | 0.6 ± 0.0 | | 0.3 ± 0.0 | | | | 4.0 ± 0.4 | | | 1.2 ± 0.1 | | | | | | | | ArcA/ArcB system, CRP-cAMP complex,  Mlc, Fis | | | | | | | | | |  |
| ***galP*** | | | Galactose MFS transporter | 30.5 ± 0.9 | | | | 12.7 ± 0.2 | | 0.9 ± 0.1 | | | | 11.9 ± 1.5 | | | 25.4 ± 0.6 | | | | | | | | CRP-cAMP complex, GalS, GalR | | | | | | | | | |  |
| ***mglB*** | | | Galactose ABC transporter | 58.9 ± 1.1 | | | | 6.7 ± 0.6 | | 2.5 ± 0.5 | | | | 119.9 ± 24.5 | | | 68.8 ± 1.7 | | | | | | | | CRP-cAMP complex, GalS, GalR, FlhDC | | | | | | | | | |  |
| ***lamB*** | | | Maltose/maltodextrin transport system | 1.6 ± 0.1 | | | | 1.1 ± 0.2 | | 1.0 ± 0.0 | | | | 34.0 ± 0.3 | | | 14.2 ± 0.7 | | | | | | | | CRP-cAMP complex, MalT | | | | | | | | | |  |
| ***ompF*** | | | Outer membrane porin | 0.1 ± 0.0 | | | | 0.3 ± 0.0 | | 0.9 ± 0.1 | | | | 1.8 ± 0.1 | | | 1.6 ± 0.1 | | | | | | | | CRP-cAMP complex, Ihf, CpxR, Lpr, EnvY,  FurNIL, OmpR | | | | | | | | | |  |
| ***malE*** | | | Maltose ABC transporter | 1.1 ± 0.0 | | | | 1.1 ± 0.1 | | 0.3 ± 0.0 | | | | 25.2 ± 2.7 | | | 14.4 ± 4.41 | | | | | | | | CRP-cAMP complex, MalT, CreBC | | | | | | | | | |  |
| **Glycolysis, gluconeogenesis and anaplerosis** | | | | | | | | | | | | | | | | | | | | | | | | | | | |  | | | | | | | |
| ***glk*** | | | Glucokinase | 2.1 ± 0.0 | | | | 1.6 ± 0.2 | | 1.1 ± 0.1 | | | | 2.4 ± 0.4 | | | 1.7 ± 0.1 | | | | | | | Cra (FruR) | | | | | | | | |  | | |
| ***pgi*** | | | Phosphoglucose isomerase | 2.7 ± 0.1 | | | | 2.3 ± 0.1 | | 0.9 ± 0.1 | | | | 3.1 ± 0.0 | | | 2.9 ± 0.0 | | | | | | | CsrA/CsrB system | | | | | | | | |  | | |
| ***pfkA*** | | | Phosphofructokinase-1 | 0.6 ± 0.1 | | | | 0.5 ± 0.0 | | 0.8 ± 0.0 | | | | 1.2 ± 0.2 | | | 0.4 ± 0.4 | | | | | | | Cra (FruR), CsrA/CsrB system | | | | | | | | |  | | |
| ***pfkB*** | | | Phosphofructokinase-2 | 1.0 ± 0.0 | | | | 0.7 ± 0.1 | | 2.9 ± 0.3 | | | | 4.0 ± 0.7 | | | 1.6 ± 0.3 | | | | | | | Uknown | | | | | | | | |  | | |
| ***fbaA*** | | | Fructose biphosphate aldolase, class II | 0.6 ± 0.0 | | | | 0.3 ± 0.0 | | 0.5 ± 0.0 | | | | 1.9 ± 0.0 | | | 0.6 ± 0.0 | | | | | | | Uknown | | | | | | | | |  | | |
| ***fbaB*** | | | Fructose biphosphate aldolase, class I | 2.1 ± 0.0 | | | | 0.9 ± 0.0 | | 0.9 ± 0.1 | | | | 1.8 ± 0.0 | | | 0.7 ± 0.0 | | | | | | | Uknown | | | | | | | | |  | | |
| ***tpiA*** | | | Triose phosphate isomerase | 0.8 ± 0.2 | | | | 0.8 ± 0.2 | | 0.4 ± 0.0 | | | | 1.1 ± 0.0 | | | 0.9 ± 0.0 | | | | | | | CsrA/CsrB system | | | | | | | | |  | | |
| ***gapA*** | | | Glyceraldehyde-3-phosphate dehydrogenase | 0.6 ± 0.0 | | | | 1.0 ± 0.1 | | 0.8 ± 0.1 | | | | 2.7 ± 0.6 | | | 0.9 ± 0.2 | | | | | | | CRP-cAMP complex | | | | | | | | |  | | |
| ***gapC-1*** | | | Glyceraldehyde-3-phosphate dehydrogenase C, truncated | 1.0 ± 0.0 | | | | 0.8 ± 0.0 | | 0.4 ± 0.1 | | | | 0.3 ± 0.1 | | | 0.2 ± 0.0 | | | | | | | FNR | | | | | | | | |  | | |
| ***gapC-2*** | | | Glyceraldehyde-3-phophate dehydrogenase C, truncated | 1.2 ± 0.0 | | | | 0.7 ± 0.0 | | 0.3 ± 0.0 | | | | 0.4 ± 0.0 | | | 0.3 ± 0.0 | | | | | | | FNR | | | | | | | | |  | | |
| ***pgk*** | | | Phosphoglycerate kinase | 1.0 ± 0.0 | | | | 0.7 ± 0.0 | | 0.8 ± 0.1 | | | | 1.3 ± 0.4 | | | 0.6 ± 0.0 | | | | | | | CRP-cAMP complex, Cra (FruR) | | | | | | | | |  | | |
| ***gpmA*** | | | Phosphoglycerate mutase 1 | 4.5 ± 0.4 | | | | 1.7 ± 0.1 | | 1.6 ± 0.4 | | | | 6.2 ± 1.9 | | | 4.9 ± 1.1 | | | | | | | Fur | | | | | | | | |  | | |
| ***gpmB*** | | | Putative β-phosphoglucomutase | 1.6 ± 0.2 | | | | 0.8 ± 0.2 | | 1.6 ± 0.1 | | | | 2.8 ± 0.2 | | | 1.0 ± 0.0 | | | | | | | Unknown | | | | | | | | |  | | |
| ***eno*** | | | Enolase | 0.4 ± 0.0 | | | | 0.6 ± 0.0 | | 0.9 ± 0.3 | | | | 0.9 ± 0.1 | | | 0.5 ± 0.1 | | | | | | | CsrA/CsrB system | | | | | | | | |  | | |
| ***pykA*** | | | Pyruvate kinase II | 0.7 ± 0.1 | | | | 0.3 ± 0.0 | | 0.3 ± 0.0 | | | | 0.8 ± 0.1 | | | 0.3 ± 0.0 | | | | | | | ArcA/ArcB system, FNR | | | | | | | | |  | | |
| ***pykF*** | | | Pyruvate kinase I | 1.2 ± 0.0 | | | | 0.6 ± 0.0 | | 0.8 ± 0.0 | | | | 1.7 ± 0.1 | | | 1.0 ± 0.2 | | | | | | | Cra (FruR), CsrA/CsrB system | | | | | | | | |  | | |
| ***fbp*** | | | Fructose 1,6 biphosphate | 2.2 ± 0.3 | | | | 0.9 ± 0.2 | | 2.2 ± 0.5 | | | | 5.1 ± 0.2 | | | 3.8 ± 0.2 | | | | | | | CsrA/CsrB system | | | | | | | | |  | | |
| ***ppsA*** | | | Phosphoenolpyruvate synthase | 4.4 ± 0.7 | | | | 2.5 ± 0.2 | | | | 5.5 ± 0.6 | | 6.5 ± 0.9 | | | | 7.0 ± 1.2 | | | | | | Cra (FruR), CsrA/CsrB system | | | | | | |  | | | | |
| ***pckA*** | | | Phosphoenolpyruvate carboxykinase | 3.4 ± 0.1 | | | | 2.9 ± 0.7 | | | | 1.0 ± 0.1 | | 4.9 ± 0.3 | | | | 1.2 ± 0.1 | | | | | | Cra (FruR) | | | | | | |  | | | | |
| ***maeB*** | | | Malic enzyme, NADP-linked | 3.1 ± 0.0 | | | | 0.8 ± 0.1 | | | | 1.3 ± 0.1 | | 7.3 ± 1.1 | | | | 1.9 ± 0.2 | | | | | | Unknown | | | | | | |  | | | | |
| ***sfcA*** | | | Malic enzyme, NAD-linked | 2.0 ± 0.1 | | | | 1.0 ± 0.1 | | | | 1.6 ± 0.3 | | 4.0 ± 0.3 | | | | 1.3 ± 0.1 | | | | | | Unknown | | | | | | |  | | | | |
| ***ppc*** | | | Phosphoenolpyruvate carboxylase | 0.4 ± 0.0 | | | | 0.3 ± 0.0 | | | | 0.6 ± 0.0 | | 0.5 ± 0.0 | | | | 0.2 ± 0.0 | | | | | | Unknown | | | | | | |  | | | | |
| ***Pentoses phosphate and Entner-Doudoroff pathways*** | | | | | | | | | | | | | | | | | | | | | | | | | |  | | | | | | | | | |
| ***zwf*** | | | Glucose 6-phophate dehydrogenase | 1.3 ± 0.0 | | | | 1.0 ± 0.0 | | | | 1.8 ± 0.0 | | 1.6 ± 0.1 | | | | 0.5 ± 0.1 | | | | | | SoxS, Rob, MarA | | | | | | |  | | | | |
| ***gnd*** | | | 6-phophogluconate dehydrogenase | 0.5 ± 0.1 | | | | 0.3 ± 0.1 | | | | 1.3 ± 1.1 | | 1.5 ± 0.3 | | | | 0.7 ± 0.1 | | | | | | GadE | | | | | | |  | | | | |
| ***rpe*** | | | Ribulose phosphate 3-epimerase | 0.8 ± 0.0 | | | | 0.5 ± 0.0 | | | | 1.1 ± 0.1 | | 1.5 ± 0.0 | | | | 0.8 ± 0.1 | | | | | | Unknown | | | | | | |  | | | | |
| ***rpiA*** | | | Ribose-5- phosphate isomerase | 1.1 ± 0.1 | | | | 1.0 ± 0.1 | | | | 1.2 ± 0.2 | | 2.7 ± 0.5 | | | | 2.2 ± 0.3 | | | | | | Unknown | | | | | | |  | | | | |
| ***tktA*** | | | Transketolase I | 0.6 ± 0.1 | | | | 1.2 ± 0.1 | | | | 65.8 ± 8.7 | | 162.6 ± 27.8 | | | | 75.1 ± 9.2 | | | | | | RpoS | | | | | | |  | | | | |
| ***tktB*** | | | Transketolase II | 2.9 ± 0.1 | | | | 0.7 ± 0.0 | | | | 0.1 ± 0.0 | | 2.7 ± 0.4 | | | | 2.2 ± 0.2 | | | | | | CreB/CreC system | | | | | | |  | | | | |
| ***talA*** | | | Transaldolase A | 5.7 ± 0.2 | | | | 1.4 ± 0.1 | | | | 1.0 ± 0.0 | | 3.8 ± 0.0 | | | | 2.8 ± 0.4 | | | | | | CreB/CreC system | | | | | | |  | | | | |
| ***talB*** | | | Transaldolase B | 1.4 ± 0.3 | | | | 0.9 ± 0.1 | | | | 0.9 ± 0.2 | | 2.4 ± 0.4 | | | | 1.1 ± 0.0 | | | | | | Unknown | | | | | | |  | | | | |
| ***eda*** | | | 2-keto-3-deoxy-6-phophogluconate aldolase | 0.6 ± 0.1 | | | | 0.6 ± 0.1 | | | | 1.2 ± 0.0 | | 1.6 ± 0.1 | | | | 0.6 ± 0.0 | | | | | | Cra (FruR), GntR, PhoB, KdgR | | | | | | |  | | | | |
| ***edd*** | | | Phosphogluconate dehydratase | 0.4 ± 0.0 | | | | 0.5 ± 0.0 | | | | 0.6 ± 0.0 | | 0.6 ± 0.0 | | | | 0.2 ± 0.0 | | | | | | Cra (FruR), GntR | | | | | | |  | | | | |
| **Pyruvate dissimilation and acetate production** | | | | | | | | | | | | | | | | | | | | | | | | | |  | | | | | | | | | |
| ***pflB*** | | | Pyruvate fomate-lyase, subunit | 0.4 ± 0.1 | | | | 0.5 ± 0.0 | | | | 0.7 ± 0.2 | | 0.3 ± 0.0 | | | | 0.2 ± 0.0 | | | | | | ArcA/ArcB system, CRP-cAMP complex, FNR, NarL | | | | | | |  | | | | |
| ***pflD*** | | | Formate acetyltransferase 2 | 1.6 ± 0.5 | | | | 1.0 ± 0.2 | | | | 2.1 ± 0.4 | | 3.4 ± 0.0 | | | | 0.9 ± 0.2 | | | | | | Unknown | | | | | | |  | | | | |
| ***aceE*** | | | Pyruvate dehydrogenase complex, subunit | 2.9 ± 0.0 | | | | 0.9 ± 0.2 | | | | 5.5 ± 0.8 | | 3.7 ± 0.2 | | | | 0.7 ± 0.0 | | | | | | ArcA/ArcB system, FNR | | | | | | |  | | | | |
| ***aceF*** | | | Pyruvate dehydrogenase complex, subunit | 1.4 ± 0.0 | | | | 0.6 ± 0.2 | | | | 3.3 ± 0.8 | | 2.3 ± 0.4 | | | | 1.6 ± 0.2 | | | | | | ArcA/ArcB system, FNR | | | | | | |  | | | | |
| ***poxB*** | | | Pyruvate oxidase | 3.5 ± 0.2 | | | | 1.2 ± 0.4 | | | | 1.1 ± 0.0 | | 2.3 ± 0.1 | | | | 2.3 ± 0.1 | | | | | | MarA, SoxS | | | | | | |  | | | | |
| ***pta*** | | | Phosphate acetyltransferase | 0.5 ± 0.0 | | | | 0.3 ± 0.0 | | | | 1.1 ± 0.0 | | 0.6 ± 0.1 | | | | 1.3 ± 0.2 | | | | | | ArcA/ArcB system, FNR | | | | | | |  | | | | |
| ***ackA*** | | | Acetate kinase A | 0.5 ± 0.0 | | | | 0.3 ± 0.1 | | | | 0.6 ± 0.0 | | 0.4 ± 0.1 | | | | 0.1 ± 0.0 | | | | | | ArcA/ArcB system, FNR | | | | | | |  | | | | |
| ***acs*** | | | Acetyl-CoA synthase | 6.5 ± 0.4 | | | | 1.4 ± 0.5 | | | | 3.0 ± 0.6 | | 111.8 ± 2.7 | | | | 96.4 ± 2.8 | | | | | | CRP-cAMP complex, Fis, Ihf | | | | | | |  | | | | |
| ***actP (yjcG)*** | | | Acetate/glyoxylate permease | 6.9 ± 0.1 | | | | 1.2 ± 0.2 | | | | 3.1 ± 0.2 | | 55.6 ± 13.7 | | | | 90.8 ± 14.6 | | | | | | CRP-cAMP complex, Fis, Ihf | | | | | | |  | | | | |
| ***ldhA*** | | | D-lactate dehydrogenase | 4.9 ± 0.1 | | | | 8.2 ± 1.7 | | | | 1.4 ± 0.3 | | 1.0 ± 0.1 | | | | 0.9 ± 0.1 | | | | | | ArcA/ArcB system, | | | | | | |  | | | | |
| ***adhE*** | | | Alcohol/acetaldehyde dehydrogenase | 0.5 ± 0.0 | | | | 0.8 ± 0.1 | | | | 0.1 ± 0.0 | | 0.4 ± 0.0 | | | | 0.2 ± 0.0 | | | | | | FNR, NarL, Cra (FruR). | | | | | | |  | | | | |
| ***aceE*** | | | Pyruvate dehydrogenase subunit | 2.9 ± 0.0 | | | | 0.9 ± 0.2 | | | | 5.5 ± 0.8 | | 3.7 ± 0.2 | | | | 0.7 ± 0.0 | | | | | | ArcA/ArcB system, FNR | | | | | | |  | | | | |
| ***aceF*** | | | Pyruvate dehydrogenase subunit | 1.4 ± 0.0 | | | | 0.5 ± 0.2 | | | | 3.3 ± 0.8 | | 2.3 ± 0.4 | | | | 1.6 ± 0.2 | | | | | | ArcA/ArcB system, FNR | | | | | | |  | | | | |
| **TCA cycle and glyoxylate shunt** | | | | | | | | | | | | | | | | | | | | | | | | | |  | | | | | | | | | |
| ***gltA*** | | | Citrate synthase | 5.0 ± 0.1 | | | | 1.4 ± 0.1 | | | | 2.3 ± 0.1 | | 8.8 ± 1.9 | | | | | | 6.8 ± 0.2 | | | | ArcA/ArcB system, Ihf, CRP-cAMP complex | | | | | | | | | | | |
| ***acnA*** | | | Aconitase | 6.0 ± 0.5 | | | | 1.0 ± 0.3 | | | | 3.8 ± 0.6 | | 22.6 ± 1.1 | | | | | | 15.3 ± 0.3 | | | | ArcA/ArcB system, CRP-cAMP complex,  FNR, Cra (FruR) | | | | | | | | | | | |
| ***acnB*** | | | Aconitase B | 1.2 ± 0.2 | | | | 0.4 ± 0.1 | | | | 3.6 ± 0.0 | | 12.7 ± 2.3 | | | | | | 9.4 ± 0.3 | | | | ArcA/ArcB system, CRP-cAMP complex,  Fis, Cra (FruR) | | | | | | | | | | | |
| ***icdA*** | | | Isocitrate dehydrogenase | 1.3 ± 0.1 | | | | 0.3 ± 0.1 | | | | 2.9 ± 0.9 | | 7.0 ± 2.4 | | | | | | 3.5 ± 0.2 | | | | ArcA/ArcB system, Cra (FruR) | | | | | | | | | | | |
| ***sucA*** | | | 2-oxoglutarate dehydrogenase, subunit | 4.6 ± 0.5 | | | | 1.0 ± 0.2 | | | | 4.3 ± 0.0 | | 18.5 ± 3.3 | | | | | | 11.8 ± 0.8 | | | | ArcA/ArcB system, FNR, Ihf | | | | | | | | | | | |
| ***sucB*** | | | 2-oxoglutarate dehydrogenase, subunit | 2.9 ± 0.5 | | | | 0.6 ± 0.1 | | | | 4.6 ± 0.2 | | 30.0 ± 1.9 | | | | | | 10.3 ± 1.0 | | | | ArcA/ArcB system, FNR, Ihf | | | | | | | | | | | |
| ***sucC*** | | | Succinyl-CoA synthase, β subunit | 3.8 ± 0.6 | | | | 0.8 ± 0.0 | | | | 2.7 ± 0.0 | | 27.1 ± 2.1 | | | | | | 9.6 ± 0.0 | | | | ArcA/ArcB system, FNR, Ihf | | | | | | | | | | | |
| ***sucD*** | | | Succinyl-CoA synthase, α subunit | 2.6 ± 0.2 | | | | 0.5 ± 0.0 | | | | 3.7 ± 0.4 | | 43.1 ± 1.7 | | | | | | 14.6 ± 1.4 | | | | ArcA/ArcB system, FNR, Ihf | | | | | | | | | | | |
| ***sdhA*** | | | Succinate dehydrogenase, subunit | 27.0 ± 2.4 | | | | 4.2 ± 0.4 | | | | 15.9 ± 0.5 | | 171.9 ± 35.1 | | | | | | 74.8 ± 12.8 | | | | ArcA/ArcB system, CRP-cAMP complex,  FNR, Fur | | | | | | | | | | | |
| ***sdhB*** | | | Succinate dehydrogenase, subunit | 10.4 ± 2.5 | | | | 1.9 ± 0.4 | | | | 13.8 ± 1.0 | | 137.8 ± 8.8 | | | | | | 39.5 ± 7.7 | | | | ArcA/ArcB system, CRP-cAMP complex,  FNR, Fur | | | | | | | | | | | |
| ***sdhC*** | | | Succinate dehydrogenase, membrane-bound subunit | 22.3 ± 3.7 | | | | 3.4 ± 0.1 | | | | 12.4 ± 1.7 | | 66.7 ± 1.3 | | | | | | 55.4 ± 10.3 | | | | ArcA/ArcB system, CRP-cAMP complex,  FNR, Fur | | | | | | | | | | | |
| ***sdhD*** | | | Succinate dehydrogenase, membrane-bound subunit | 30.2 ± 3.8 | | | | 4.2 ± 0.1 | | | | 16.5 ± 0.4 | | 117.0 ± 2.3 | | | | | | 56.8 ± 5.3 | | | | ArcA/ArcB system, CRP-cAMP complex,  FNR, Fur | | | | | | | | | | | |
| ***fumA*** | | | Fumarase A | 12.4 ± 2.0 | | | | 1.2 ± 0.0 | | | 4.0 ± 1.6 | | | 25.9 ± 2.3 | | | | | | 5.4 ± 0.4 | | | | ArcA/ArcB system, FNR | | | | | | | | | | | |
| ***fumB*** | | | Fumarase B | 0.3 ± 0.0 | | | | 0.4 ± 0.0 | | | 0.2 ± 0.0 | | | 0.2 ± 0.0 | | | | | | 0.1 ± 0.0 | | | | ArcA/ArcB system, Fur, Fis, FNR | | | | | | | | | | | |
| ***mdh*** | | | Malate dehydrogenase | 1.5 ± 0.3 | | | | 0.4 ± 0.0 | | | 3.7 ± 0.3 | | | 16.7 ± 1.1 | | | | | | 6.7 ± 0.6 | | | | ArcA/ArcB system, CRP-cAMP complex,  FlhDC | | | | | | | | | | | |
| ***aceA*** | | | Isocitrate lyase, subunit | 0.5 ± 0.1 | | | | 0.8 ± 0.0 | | | 1.8 ± 0.1 | | | 30.9 ± 10.2 | | | | | | 30.4 ± 10.8 | | | | ArcA/ArcB system, CRP-cAMP complex,  IcIR, Ihf, Cra (FruR) | | | | | | | | | | | |
| ***aceB*** | | | Malate synthase A | 0.4 ± 0.0 | | | | 1.0 ± 0.0 | | | 3.8 ± 0.6 | | | 50.8 ± 6.9 | | | | | | 47.5 ± 7.4 | | | | ArcA/ArcB system, CRP-cAMP complex,  IcIR, Ihf, Cra (FruR) | | | | | | | | | | | |
| ***glcB*** | | | Malate synthase G | 3.9 ± 0.2 | | | | 0.6 ± 0.1 | | | 0.8 ± 0.2 | | | 5.9 ± 0.7 | | | | | | 3.0 ± 0.3 | | | | ArcA/ArcB system, Ihf, GlcC | | | | | | | | | | | |
| ***Regulators*** | | | | | | | | | | | | | | | | | | | | | | | | | | |  | | | | | | | | |
| ***arcA*** | | | Transcriptional dual regulator | 1.8 ± 0.0 | | | | 1.6 ± 0.0 | | 2.5 ± 0.4 | | | | 2.2 ± 0.1 | | | 2.0 ± 0.0 | | | | | | | FNR | | | | | | | |  | | | |
| ***arcB*** | | | Sensor kinase phosphotransferase | 1.0 ± 0.2 | | | | 1.0 ± 0.1 | | 0.6 ± 0.0 | | | | 1.1 ± 0.3 | | | 0.7 ± 0.1 | | | | | | | Unknown | | | | | | | |  | | | |
| ***Cra (fruR)*** | | | Transcriptional dual regulator | 1.5 ± 0.0 | | | | 1.4 ± 0.3 | | 0.8 ± 0.2 | | | | 0.6 ± 0.0 | | | 0.4 ± 0.0 | | | | | | | Unknown | | | | | | | |  | | | |
| ***mlc*** | | | Transcriptional repressor | 1.9 ± 0.1 | | | | 1.0 ± 0.2 | | 0.8 ± 0.0 | | | | 4.3 ± 0.6 | | | 3.0 ± 0.5 | | | | | | | Mlc, CRP-cAMP complex | | | | | | | |  | | | |
| ***crp*** | | | Transcriptional dual regulator | 1.0 ± 0.3 | | | | 0.5 ± 0.0 | | 1.0 ± 0.1 | | | | 1.4 ± 0.2 | | | 0.8 ± 0.1 | | | | | | | CRP-cAMP complex | | | | | | | |  | | | |
| ***fnr*** | | | Transcriptional dual regulator | 3.5 ± 0.1 | | | | 2.8 ± 0.0 | | 2.4 ± 0.2 | | | | 5.0 ± 0.2 | | | 4.1 ± 0.2 | | | | | | | ArcA/ArcB system, FNR | | | | | | | |  | | | |
| ***csrA*** | | | Carbon storage regulator | 1.5 ± 0.5 | | | | 0.8 ± 0.2 | | 1.1 ± 0.1 | | | | 3.0 ± 0.2 | | | 1.4 ± 0.3 | | | | | | | Unknown | | | | | | | |  | | | |
| ***csrB*** | | | Noncoding RNA, CsrA antagonist | 4.4 ± 0.6 | | | | 3.3 ± 0.3 | | 0.3 ± 0.0 | | | | 4.8 ± 0.7 | | | 3.3 ± 0.6 | | | | | | | Unknown | | | | | | | |  | | | |
| ***creB*** | | | Transcriptional regulator | 1.2 ± 0.0 | | | | 0.7 ± 0.0 | | 2.9 ± 0.8 | | | | 2.9 ± 1.0 | | | 1.4 ± 0.1 | | | | | | | Unknown | | | | | | | |  | | | |
| ***creC*** | | | Sensor kinase phosphotransferase | 0.8 ± 0.0 | | | | 0.6 ± 0.1 | | 2.5 ± 0.3 | | | | 1.7 ± 0.5 | | | 0.9 ± 0.1 | | | | | | | Unknown | | | | | | | |  | | | |
| ***ompR*** | | | Transcriptional dual regulator | 0.9 ± 0.2 | | | | 0.5 ± 0.1 | | 1.7 ± 0.3 | | | | 2.1 ± 0.0 | | | 1.2 ± | | | | | | | Ihf, CRP-cAMP complex | | | | | | | |  | | | |
| ***tyrR*** | | | Transcriptional dual regulator | 2.0 ± 0.1 | | | | 1.3 ± 0.0 | | 1.0 ± 0.1 | | | | 2.1 ± 0.2 | | | 0.7 ± 0.2 | | | | | | | TyrR | | | | | | | |  | | | |
| ***trpR*** | | | Transcriptional repressor | 1.3 ± 0.1 | | | | 1.5 ± 0.1 | | 3.3 ± 0.3 | | | | 3.5 ± 0.3 | | | 2.2 ± 0.1 | | | | | | | TrpR | | | | | | | |  | | | |
| ***rpoS*** | | | Sigma S factor | 6.1 ± 0.7 | | | | 2.1 ± 0.5 | | 3.9 ± 0.8 | | | | 9.6 ± 0.1 | | | 3.8 ± 0.2 | | | | | | | CRP-cAMP complex, GadX | | | | | | | |  | | | |
| ***rpoD*** | | | Sigma 70 factor | 2.0 ± 0.2 | | | | 0.6 ± 0.0 | | 2.6 ± 0.4 | | | | 2.2 ± 0.2 | | | 0.6 ± 0.1 | | | | | | | Unknown | | | | | | | |  | | | |
| ***rpoE*** | | | Sigma E factor | 1.8 ± 0.4 | | | | 0.7 ± 0.1 | | 0.9 ± 0.1 | | | | 1.3 ± 0.1 | | | 1.3 ± 0.1 | | | | | | | CpxR | | | | | | | |  | | | |
| ***rpoH*** | | | Sigma 32 factor | 2.4 ± 0.2 | | | | 1.0 ± 0.0 | | 2.3 ± 0.2 | | | | 3.8 ± 0.6 | | | 0.9 ± 0.1 | | | | | | | CRP-cAMP complex, CytR, DNA-ATP | | | | | | | |  | | | |
| ***glcC*** | | | Transcriptional dual regulator | 51.0 ± 9.4 | | | | 5.2 ± 0.4 | | 19.4 ± 4.6 | | | | 63.1 ± 1.2 | | | 29.8 ± 7.5 | | | | | | | ArcA/ArcB system, Ihf, GlcC, CRP-cAMP complex. | | | | | | | |  | | | |
| ***gltF*** | | | Regulator of *gltBDF* operon | 0.8 ± 0.0 | | | | 0.7 ± 0.1 | | 2.5 ± 0.4 | | | | 1.1 ± 0.2 | | | 0.4 ± 0.1 | | | | | | | Lpr, GadE, Nac | | | | | | | |  | | | |
| **Aromatic amino acid pathways and transport** | | | | | | | | | | | | | | | | | | | | | | | | | | |  | | | | | | | | |
| ***aroF*** | | | DAHP synthase, sensitive to feedback inhibition by Tyr | 1.4 ± 0.2 | | | | 1.3 ± 0.4 | | 2.1 ± 0.1 | | | | 0.6 ± 0.1 | | | | | | 0.1 ± 0.0 | | | | TyrR | | | | | | | | | | |  |
| ***aroG*** | | | DAHP synthase: aroG (wt) sensitive to feedback inhibition by Phe; aroG (fbr) feedback resistant | 0.7 ± 0.0 | | | | 0.6 ± 0.1 | | 4.4 ± 0.1 | | | | 10.6 ± 1.1 | | | | | | 10.0 ± 0.3 | | | | Attenuation (*aroG*, wt) or  inducible by IPTG (*aroG*, fbr) | | | | | | | | | | |  |
| ***aroH*** | | | DAHP synthase, sensitive to feedback inhibition by Trp | 1.4 ± 0.2 | | | | 1.0 ± 0.1 | | 1.6 ± 0.1 | | | | 1.2 ± 0.4 | | | | | | 2.1 ± 0.7 | | | | TrpR | | | | | | | | | | |  |
| ***aroB*** | | | 3-dehydroquinate synthase | 0.7 ± 0.2 | | | | 0.5 ± 0.1 | | 1.4 ± 0.1 | | | | 1.1 ± 0.2 | | | | | | 0.5 ± 0.0 | | | | Unknown | | | | | | | | | | |  |
| ***aroD*** | | | 3-dehydroquinate dehydratase | 0.5 ± 0.0 | | | | 0.4 ± 0.1 | | 0.8 ± 0.0 | | | | 1.1 ± 0.2 | | | | | | 25.6 ± 1.6 | | | | Unknown | | | | | | | | | | |  |
| ***aroE*** | | | Shikimate dehydrogenase I | 1.8 ± 0.0 | | | | 0.7 ± 0.1 | | 0.9 ± 0.0 | | | | 0.7 ± 0.0 | | | | | | 0.3 ± 0.0 | | | | Unknown | | | | | | | | | | |  |
| ***ydiB*** | | | Shikimate dehydrogenase II | 1.7 ± 0.2 | | | | 0.9 ± 0.1 | | 7.4 ± 0.9 | | | | 9.7 ± 0.3 | | | | | | 7.2 ± 1.9 | | | | Unknown | | | | | | | | | | |  |
| ***aroL*** | | | Shikimate kinase II | 1.3 ± 0.0 | | | | 0.6 ± 0.0 | | 2.0 ± 0.1 | | | | 0.7 ± 0.0 | | | | | | 0.7 ± 0.0 | | | | TyrR, TrpR | | | | | | | | | | |  |
| ***aroK*** | | | Shikimate kinase I | 1.0 ± 0.1 | | | | 0.9 ± 0.1 | | 1.5 ± 0.3. | | | | 0.8 ± 0.2 | | | | | | 0.6 ± 0.0 | | | | Unknown | | | | | | | | | | |  |
| ***aroM*** | | | Putative protein | 1.6 ± 0.3 | | | | 0.5 ± 0.0 | | 1.5 ± 0.0 | | | | 0.8 ± 0.0 | | | | | | 0.5 ± 0.1 | | | | Unknown | | | | | | | | | | |  |
| ***aroA*** | | | 5-enol-pyruvylshikimate-3-phosphate synthase | 0.4 ± 0.0 | | | | 0.4 ± 0.1 | | 1.3 ± 0.0 | | | | 1.7 ± 0.3 | | | | | | 0.5 ± 0.0 | | | | Lpr | | | | | | | | | | |  |
| ***aroC*** | | | Chorismate synthase | 1.1 ± 0.1 | | | | 0.9 ± 0.0 | | 2.2 ± 0.0 | | | | 3.0 ± 0.1 | | | | | | 1.2 ± 0.1 | | | | Unknown | | | | | | | | | | |  |
| ***pheA* wt** | | | Chorismate mutase-prephenate dehydratase | 2.2 ± 0.1 | | | | 1.3 ± 0.3 | | 0.4 ± 0.0 | | | | 0.5 ± 0.0 | | | | | | 0.8 ± 0.1 | | | | Attenuation | | | | | | | | | | |  |
| ***pheA-ev2*** | | | Chorismate mutase-prephenate dehydratase, fbr to Phe | 1.5 ± 0.2 | | | | 1.0 ± 0.2 | | 92.2 ± 19.7 | | | | 126.4 ± 9.9 | | | | | | 39.9 ± 0.8 | | | | Inducible by IPTG | | | | | | | | | | |  |
| ***aspC*** | | | Aspartate transaminase | 0.3 ± 0.0 | | | | 0.3 ± 0.0 | | 0.9 ± 0.2 | | | | 1.4 ± 0.1 | | | | | | 1.3 ± 0.0 | | | | Unkown | | | | | | | | | | |  |
| ***ilvE*** | | | Branch chain amino acid aminotransferase | 1.1 ± 0.2 | | | | 0.9 ± 0.0 | | 1.0 ± 0.1 | | | | 1.2 ± 0.1 | | | | | | 0.6 ± 0.0 | | | | Ihf, Lpr | | | | | | | | | | |  |
| ***tyrA*** | | | Chorismate mutase-prephenate dehydrogenase | 1.3 ± 0.1 | | | | 1.1 ± 0.2 | | 2.7 ± 0.2 | | | | 1.1 ± 0.1 | | | | | | 0.6 ± 0.0 | | | | TyrR | | | | | | | | | | |  |
| ***trpA*** | | | Tryptophan synthase, α subunit | 0.5 ± 0.1 | | | | 0.3 ± 0.1 | | 0.4 ± 0.0 | | | | 0.5 ± 0.1 | | | | | | 0.2 ± 0.0 | | | | TrpR, attenuation | | | | | | | | | | |  |
| ***trpE*** | | | Antranilate synthase, component I | 1.0 ± 0.1 | | | | 0.6 ± 0.0 | | 0.2 ± 0.0 | | | | 0.2 ± 0.0 | | | | | | 0.1 ± 0.0 | | | | TrpR, attenuation | | | | | | | | | | |  |
| ***aroP*** | | | Phe/Tyr/Trp transporter | 1.5 ± 0.2 | | | | 0.7 ± 0.1 | | 2.3 ± 0.1 | | | | 1.0 ± 0.0 | | | | | | 0.3 ± 0.0 | | | | TyrR | | | | | | | | | | |  |
| ***pheP*** | | | Phe transporter | 1.1 ± 0.2 | | | | 0.6 ± 0.2 | | 5.6 ± 0.6 | | | | 3.4 ± 0.5 | | | | | | 1.3 ± 0.4 | | | | Uknown | | | | | | | | | | |  |
| ***tyrP*** | | | Tyr transporter | 1.7 ± 0.2 | | | | 0.7 ± 0.1 | | 3.1 ± 0.1 | | | | 1.7 ± 0.0 | | | | | | 0.8 ± 0.0 | | | | TyrR, Ihf | | | | | | | | | | |  |
| ***mtr*** | | | Trp transporter | 0.6 ± 0.2 | | | | 0.6 ± 0.1 | | 1.8 ± 0.0 | | | | 2.4 ± 0.2 | | | | | | 1.0 ± 0.2 | | | | TrpR, TyrR, Ihf | | | | | | | | | | |  |
| ***shiA*** | | | Shikimate transporter | 1.3 ± 0.4 | | | | 0.5 ± 0.1 | | 0.3 ± 0.0 | | | | 0.3 ± 0.1 | | | | | | 0.2 ± 0.0 | | | | Uknown | | | | | | | | | | |  |
| **Nitrogen metabolism pathways** | | | | | | | | | | | | | | | | | | | | | | | | | | |  | | | | | | | | |
| ***gdhA*** | | | Glutamate dehydrogenase (NADP^+^) | 0.3 ± 0.0 | | | | 0.2 ± 0.0 | | 0.1 ± 0.0 | | | | | 0.4 ± 0.1 | | | | 0.2 ± 0.0 | | | | | Nac | | | | | | | | | | | |
| ***glnA*** | | | Glutamine synthase | 0.4 ± 0.1 | | | | 1.8 ± 0.3 | | 0.9 ± 0.1 | | | | | 0.8 ± 0.1 | | | | 0.1 ± 0.0 | | | | | CRP-cAMP complex, NtrC | | | | | | | | | | | |
| ***gltB*** | | | Glutamate synthase, large chain precursor | 0.7 ± 0.1 | | | | 0.3 ± 0.0 | | 0.8 ± 0.0 | | | | | 0.5 ± 0.0 | | | | 0.1 ± 0.0 | | | | | Lpr, GadE, Nac | | | | | | | | | | | |
| ***gltD*** | | | Glutamate synthase, small chain | 0.5 ± 0.1 | | | | 0.2 ± 0.0 | | 0.8 ± 0.1 | | | | | 0.4 ± 0.0 | | | | 0.1 ± 0.0 | | | | | Lpr, GadE, Nac | | | | | | | | | | | |

This table shows the relative transcription levels of genes grouped by metabolic pathway or process. RT–PCR values of the reference strain (JM101) were adjusted to one and used to normalize all data of PB12, PB13, JM101-ev2, PB12-ev2 and PB13-ev2. Therefore, all data in this table and Figs. 2 to 4 are reported as relative transcription levels, as compared to JM101. All results correspond to the average of six measurements of the RT–PCR expression values for each gene. Half of the values were obtained from two different cDNAs generated in independent experiments. The RT–PCR transcription values obtained for each gene differ in most of the genes by less than 30% (see Materials and methods for a more detailed explanation).
